# Extravesicular TIMP-1 is a non-invasive independent prognostic marker and potential therapeutic target in colorectal liver metastases

**DOI:** 10.1038/s41388-022-02218-9

**Published:** 2022-02-09

**Authors:** Venkatesh Sadananda Rao, Qianyu Gu, Sandra Tzschentke, Kuailu Lin, Nicole Ganig, May-Linn Thepkaysone, Fang Cheng Wong, Heike Polster, Lena Seifert, Adrian M. Seifert, Nathalie Buck, Carina Riediger, Jonas Weiße, Tony Gutschner, Susanne Michen, Achim Temme, Martin Schneider, Franziska Baenke, Jürgen Weitz, Christoph Kahlert

**Affiliations:** 1 Department of Visceral, Thoracic and Vascular Surgery, University Hospital Carl Gustav Carus, Technische Universität Dresden, Dresden, Germany; 2 Department of Medicine, Haematology/Oncology, University Hospital Frankfurt, Goethe University, Frankfurt am Main, Germany; 3 German Cancer Consortium (DKTK), German Cancer Research Centre (DKFZ), Heidelberg, Germany; 4 National Center for Tumor Diseases, Partner site Dresden, Heidelberg, Germany; 5 Junior Research Group ‘RNA Biology and Pathogenesis’, Medical Faculty, Martin-Luther University Halle-Wittenberg, Halle/Saale, Germany; 6 Department of Neurosurgery, Section of Experimental Neurosurgery and Tumour Immunology, University Hospital Carl Gustav Carus, Technische Universität Dresden, Dresden, Germany; 7 Department of General, Visceral and Transplantation Surgery, University Hospital Heidelberg, Heidelberg, Germany

**Keywords:** Predictive markers, Metastasis, Prognostic markers

## Abstract

Molecular reprogramming of stromal microarchitecture by tumour-derived extracellular vesicles (EVs) is proposed to favour pre-metastatic niche formation. We elucidated the role of extravesicular tissue inhibitor of matrix metalloproteinase-1 (TIMP1^EV^) in pro-invasive extracellular matrix (ECM) remodelling of the liver microenvironment to aid tumour progression in colorectal cancer (CRC). Immunohistochemistry analysis revealed a high expression of stromal TIMP1 in the invasion front that was associated with poor progression-free survival in patients with colorectal liver metastases. Molecular analysis identified TIMP1^EV^ enrichment in CRC-EVs as a major factor in the induction of TIMP1 upregulation in recipient fibroblasts. Mechanistically, we proved that EV-mediated TIMP1 upregulation in recipient fibroblasts induced ECM remodelling. This effect was recapitulated by human serum-derived EVs providing strong evidence that CRC release active EVs into the blood circulation of patients for the horizontal transfer of malignant traits to recipient cells. Moreover, EV-associated TIMP1 binds to HSP90AA, a heat-shock protein, and the inhibition of HSP90AA on human-derived serum EVs attenuates TIMP1^EV^-mediated ECM remodelling, rendering EV-associated TIMP1 a potential therapeutic target. Eventually, in accordance with REMARK guidelines, we demonstrated in three independent cohorts that EV-bound TIMP1 is a robust circulating biomarker for a non-invasive, preoperative risk stratification in patients with colorectal liver metastases.

## Introduction

Liver metastases are the leading cause of most colorectal cancer (CRC)-related deaths [1, 2]. Despite recent advances in precision medicine, surgery is the only therapy offering the possibility of remission for patients with hepatic metastatic disease [3]. Therefore, to achieve a good prognosis, early detection is of paramount importance, and gaining better insight into the metastatic cascade employed by tumour cells to successfully metastasise distant organs is imperative.

Primary tumours release soluble factors that condition specific sites in secondary organs in order to establish a pre-metastatic niche [4]. Tissue inhibitor of matrix metalloproteinase-1 (TIMP1), a secretory molecule, is consistently associated with the establishment of a pre-metastatic niche in the liver, leading to metastasis in CRC [5, 6]. Overexpression of TIMP1 in the stroma at the invasive front has been demonstrated in primary CRC and liver metastases (CRC liver MET) [7, 8]; however, the precise molecular function of elevated stromal TIMP1 levels on the liver microenvironment remain to be elucidated.

Since their discovery, extracellular vesicles (EVs) have garnered immense attention owing to their role in the establishment and maintenance of the tumour microenvironment (TME), including the sustenance of cell proliferation and remodelling of the extracellular matrix (ECM) [9–11]. Reports suggest that tumour cells secrete more EVs than normal cells, resulting in elevated EV concentrations in cancer patients relative to those in healthy controls [12–14]. Given their organotrophic tendencies, tumour-derived EVs travel to distant organs and interact with and modulate the ECM complex, thereby paving the way for tumour-cell infiltration. As ECM dysregulation has frequently been reported as the primary step of the invasion–metastasis cascade [15], a comprehensive investigation of tumour cell-derived EVs and their interaction with the ECM complex would be beneficial for identifying the driving factors involved in the priming of distant organs and subsequent tumour invasion.

Here, we investigated the role of CRC-derived EVs (CRC-EVs) in the remodelling of the liver microenvironment to promote liver metastasis in CRC. We demonstrated that CRC-derived extravesicular TIMP1 (TIMP1^EV^) upregulated TIMP1 levels in recipient liver fibroblasts leading to the onset of ECM remodelling; further, the evaluation of TIMP1^EV^ as an intervention target and non-invasive biomarker is warranted to lead to the development of novel treatment strategies for preventing liver metastasis in CRC.

## Results

### Invasion front-specific TIMP1 overexpression in the stroma of patients with CRC liver MET is associated with poor progression-free survival (PFS)

**Fig. 1:**
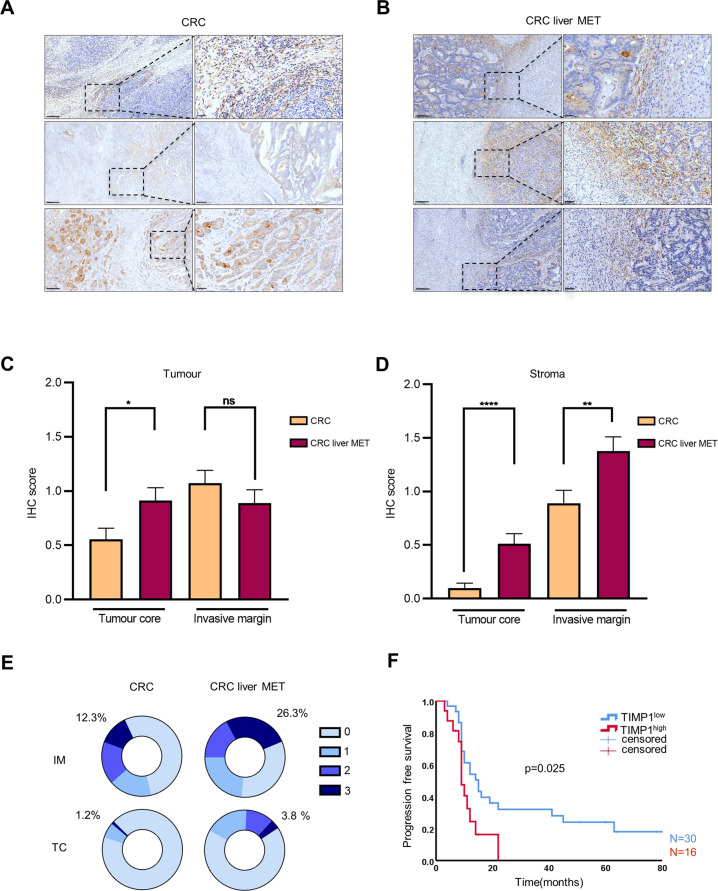
Elevated TIMP1 levels in tumour stroma of CRC liver MET. **A**, **B** Representative immunohistochemial (IHC) images showing localisation patterns of TIMP1 in CRC (*n* = 81) and CRC liver MET (*n* = 80). **C** Quantification of TIMP1 expression in the tumour core (TC) and invasive margin (IM) of the tumour as identified by IHC in CRC and CRC liver MET. **D** Quantification of TIMP1 expression in the stroma (TC and IM) as identified by IHC in CRC and CRC liver MET. **E** Percentage of tissues with TIMP1^high^ (score 3) and TIMP1^low^ (score 0–2) stroma in the IM and TC of CRC and CRC liver MET. **F** Kaplan-Meier curve depicting progression-free survival for CRC liver MET patients with TIMP1^high^ (*n* = 16) compared to TIMP1^low^ (*n* = 30) stroma in IM. Scale bars: 300 µm. P values were calculated by unpaired t-test or Log-rank (Mantel-Cox) test (**F**). *p < 0.05, **p < 0.01, ****p < 0.0001, ns not significant.

Data mining using the Oncomine portal (www.oncomine.org) [16] revealed that *TIMP1* ranks among the top 1% of overexpressed genes in tissue biopsies of CRC patients. Several studies have reported elevated TIMP1 levels in liquid and solid biopsies; however, little is known about its localisation. Therefore, we performed an immunohistochemical (IHC) analysis on a panel of 81 primary tumour and 80 metastasised liver resections from CRC patients. TIMP1 was prominently localised in the tumour core (TC) of primary tumour tissues (Figs. 1A and S1), and a high expression of TIMP1 was observed in the invasive margin (IM) of liver MET tissues (Figs. 1B and S1). Quantification of TIMP1 in the tumour region of all assessed slides showed a higher TIMP1 expression in the TC of liver MET relative to that in primary CRC tissues (Fig. 1C). However, no significant difference was observed in TIMP1 localisation in the IM of both liver MET and CRC (Fig. 1C). Interestingly, the stroma around the tumour tissues showed a preponderance of TIMP1 level in both the TC and IM of liver MET relative to that in primary CRC (Fig. 1D). These results suggested that TIMP1 is more highly expressed in the stroma of liver MET than in primary CRC.

As TIMP1 expression was substantially elevated in the stroma of liver MET, we investigated whether TIMP1 localisation in the stroma could be specific to liver MET. Based on the TIMP1 staining intensity, the stroma of CRC and CRC liver MET were categorised into TIMP1^low^ (staining score: 0–2) or TIMP1^high^ (staining score: 3). Consistently, > 26% of the analysed liver MET harboured TIMP1^high^ stroma in the IM, whereas only 12.3% of all primary CRC were categorised as TIMP1^high^ (Fig. 1E, top). Similarly, > 3.7% of liver MET analysed showed TIMP1^high^ stroma in the TC, whereas only 1.2% were categorised as TIMP1^high^ in all primary CRC (Fig. 1E, bottom). In CRC liver MET, TIMP1 was mainly overexpressed in the IM than in the TC (Fig. 1E). These results suggested that the TIMP1 overexpression observed in the stroma of patients with CRC liver MET was IM-specific. Kaplan–Meier survival analysis using a log-rank (Mantel–Cox) test for PFS showed that the TIMP1^high^ stroma in the IM (median PFS: 9 months) of patients with CRC liver MET resulted in significantly poorer PFS relative to that associated with TIMP1^low^ stroma (median PFS: 15 months, *p* = 0.025) (Fig. 1F). Collectively, our patient data demonstrated that TIMP1 overexpression in the stroma and TIMP1^high^ IM in CRC liver MET were associated with poor PFS.

### CRC-derived TIMP1^EV^ regulates TIMP1 levels in recipient fibroblasts

**Fig. 2:**
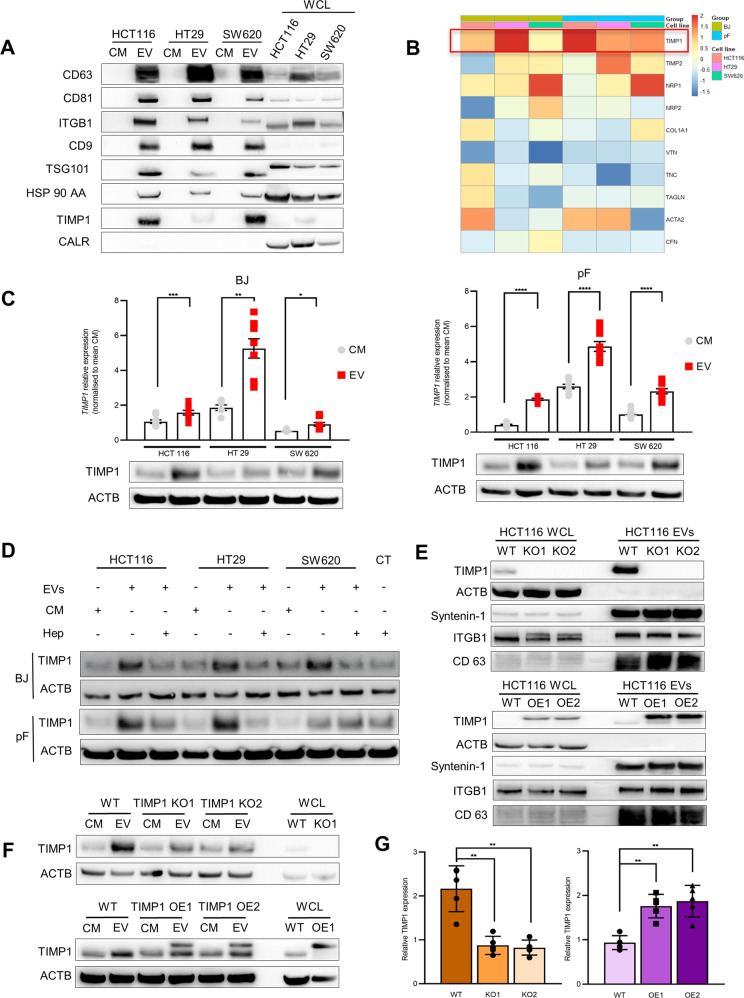
TIMP1^EV^ regulate TIMP1 levels in recipient fibroblasts. **A** Immunoblot analysis of EV proteins CD63, CD81, ITGB1, CD9, TSG101, HSP90AA, TIMP1, and calreticulin as negative control in CM and EV fractions in 3 CRC cell lines. Whole-cell lysate (WCL) used as internal control. **B** Heat map of qPCR data illustrating differential expression pattern of various ECM genes in BJ and pF fibroblasts treated with CRC-EVs for 24 h. Values were normalised to CM controls. **C** *TIMP1* mRNA levels in CM and EV treated fibroblasts (BJ and pF) (top) and corresponding TIMP1 protein levels (bottom). **D** Immunoblot of TIMP1 protein levels in BJ (top) and pF (Bottom) cells after 48 h CRC-EV and CM treatment and after a preceded heparin treatment (100 μg/ml) for 3 h. **E** Immunoblot showing TIMP1 expression in WCL and EV fraction derived from HCT116 TIMP1^KO^ clones (KO1 and KO2) (top) and HCT116 TIMP1^OE^ clones (OE1 and OE2) (bottom). Parental HCT116 cells (TIMP1^WT^) used as reference. ACTB and EV markers ITGB1, Syntenin1, and CD63 were used as loading controls. **F** Immunoblot analysis of TIMP1 expression in pFs treated with CM and EVs from TIMP1^WT^, TIMP KO1 and TIMP1 KO2 cells (top) and TIMP1^WT^, TIMP OE1, and TIMP1 OE2 cells (bottom). Beta-actin (ACTB) was used as a loading control. **G** Densitometry quantification of TIMP1 protein expression normalised to ACTB shown in (**G**) using imageJ. All experiments have been performed at least 3 times. Error bars depict mean ± SEM. *P* values were calculated by unpaired t-test: *p < 0.05, **p < 0.01, ***p < 0.001, ****p < 0.0001.

Several studies as well as our IHC data have demonstrated that the overexpression of TIMP1 occurs predominantly at the tumour invasion front in CRC and metastases [7, 8]. The tumour invasion front is defined as the interphase where tumour cells directly interact with the microenvironment of the host organ. Other than direct cell-to-cell contacts and soluble factors, there is emerging evidence that EVs play a pivotal role in this intercellular communication. Thus, we aimed to determine whether elevated TIMP1 levels in tumour stroma, relative to those in the tumour cells themselves, are mediated by CRC-EVs. Three human CRC cell lines (HCT 116, HT29, and SW620) were used to isolate EVs, which were then characterised according to the 2018 MISEV guidelines [17] using nanoparticle tracking analysis (NTA), electron microscopy (TEM), and immunoblot analysis for exosomal markers (Figs. 2A and S3A).

As a preliminary step, we assessed TIMP1 protein levels in the CRC cell lines and their corresponding EVs. Interestingly, TIMP1 was more enriched in the EVs (TIMP1^EV^) than in the corresponding parental cells (Fig. 2A). TIMP1 reportedly exhibits cytokine-like functions and engages in intracellular signalling events that result in altered gene expression and changes in cell behaviour [18]. To investigate whether TIMP1^EV^ has a similar function in this context, we employed liver-derived primary fibroblasts (pF) and BJ fibroblasts. A set of candidate genes including *TIMP1* implicated in the alteration of the ECM composition was selected for the analysis [19–21]. EV-treated fibroblasts showed a distinct gene-expression signature compared to fibroblasts treated with conditioned medium (CM) depleted of EVs (Fig. 2B). Among those candidates, we observed a 2-fold increase in relative *TIMP1* mRNA levels in all fibroblasts treated with CRC-EVs whereas the other ECM-related genes showed heterogeneous expression in the treated fibroblasts (Fig. 2C, top). Likewise, TIMP1 protein levels correlated with *TIMP1* mRNA levels and were in line with the significant upregulation of TIMP1 level observed in fibroblasts treated with CRC-EVs relative to controls (Fig. 2C, bottom). The liver is perceived to be a sex hormone-responsive organ [22]. Sex-specific differences in liver function are known to exist. Hence we assessed the sex-specific effect of CRC-EVs on the fibroblasts. Sex-based classification of CRC-EVs and the recipient fibroblasts showed no significant difference in EV-mediated TIMP1 upregulation between the sexes (Table S1 and Fig. S2). Heparin can attenuate EV uptake by recipient cells via heparan sulfate receptor blockade [23, 24]. To examine whether TIMP1 upregulation in treated fibroblasts is EV-mediated, fibroblasts were pre-treated with 100 µg/mL heparin for 3 h prior to EV exposure. We observed significant inhibition of TIMP1 upregulation upon heparin treatment of CRC-EV-treated BJ fibroblasts and pFs (Figs. 2D and S3B, C). Next, to determine the cytokine-like functions of TIMP1, human recombinant TIMP1 purified from a mouse myeloma cell line was used to stimulate pFs in increasing concentrations at different time points. RT-qPCR analysis of TIMP1 expression in the treated pFs showed that exogenous TIMP1 induces TIMP1 mRNA expression in the recipient cells, and the exogenous TIMP1 levels correlate with TIMP1 induction levels in the recipient cells (Fig. S3D–F). Taken together, these results indicated that CRC-derived TIMP1^EV^ has a cytokine-like function and induces TIMP1 RNA and protein upregulation in recipient fibroblasts.

We then assessed whether TIMP1^EV^ is selectively enriched in CRC-EVs and whether TIMP1^EV^ levels are directly affected by cellular TIMP1 levels in cancer cells. We used the CRISPR-Cas9 technique to ablate *TIMP1* expression in HCT116 cells (TIMP1^KO^), followed by analysis of selected single clones and their corresponding EVs for TIMP1 expression. We observed significant reductions in cellular TIMP1 levels in the selected knockout clones and TIMP1^EV^ in their corresponding EVs (Fig. 2E, top). Similarly, overexpression of TIMP1 (TIMP1^OE^) resulted in a 2-fold increase in cellular TIMP1 level and corresponding TIMP1^EV^ levels in HCT116 cells (Fig. 2E, bottom). Notably, modulation of TIMP1 did not alter the phenotype, proliferative capacity, or EV production of HCT 116 cells. Additionally, TEM and NTA revealed no changes in EV morphology and production (Fig. S4A–C).

To determine the effect of altered TIMP1^EV^ levels in HCT 116 EVs on recipient fibroblasts, pFs were treated with EVs (isolated from TIMP1^WT^, TIMP1^OE^, and TIMP1^KO^ cells) for 48 h. We observed a significant reduction in TIMP1 levels (> 2-fold) in the pFs treated with TIMP1^KO^ EVs relative to those after TIMP1^WT^ EV treatment (Fig. 2F, G). Similarly, there was a significant increase in TIMP1 levels (>2-fold) in the pFs treated with TIMP1^OE^ EVs relative to those after TIMP1^WT^ EV treatment (Fig. 2F, G). These results suggested that TIMP1^EV^ levels in CRC-EVs significantly regulate cellular TIMP1 expression in recipient liver pFs.

### EV-mediated TIMP1 upregulation in pFs induces ECM remodelling

**Fig. 3:**
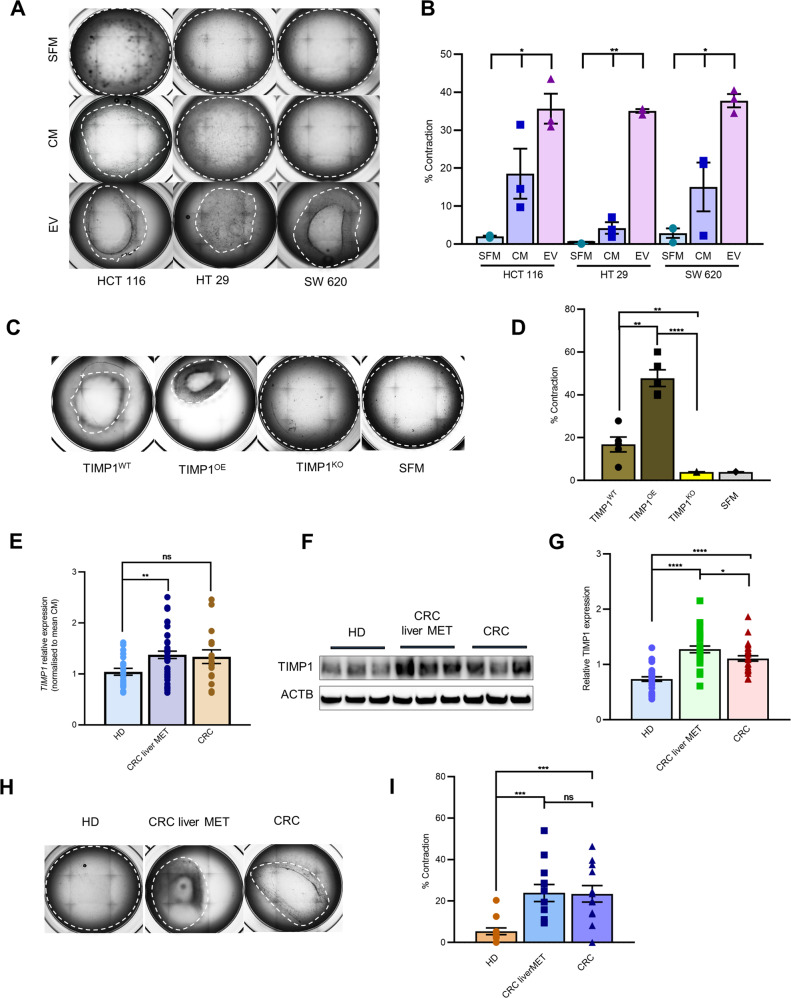
CRC patient serum-derived TIMP1^EV^ promotes ECM remodelling. **A** Representative bright field images of collagen matrigel lattices with embedded pFs treated with CRC EVs (HCT116, HT29, SW620) after 48 h (*n* = 3), white dashed line highlighting the matrix margins. **B** Percentage of contraction shown in (**A**) exerted by SFM, CM and EV treated pFs as calculated using imageJ. **C** Representative bright field images of collagen matrigel lattices with embedded pFs treated with EVs from TIMP1^OE^, TIMP1^WT^, and TIMP1^KO^ cell lines after 48 h, white dashed line highlighting the matrix margins. SFM treated pFs were used as control (*n* = 5). **D** Percentage of matrix contraction shown in (**C**) calculated using imageJ (*n* = 5). **E** *TIMP-1* mRNA expression in pFs treated with serum EVs from HD (*n* = 20), CRC liver MET (*n* = 46) and CRC (*n* = 18) using qPCR. Values were normalised to mean CM. **F** Representative Immunoblot analysis of TIMP1 expression in pFs treated with serum EVs from HD, CRC liver MET and CRC. (Shown: *n* = 3, Total: *n* = 27 per group). B-actin (ACTB) was used as a loading control. **G** Densitometry quantification of TIMP1 expression normalised to ACTB of all blots shown in (**F**) using imageJ (*n* = 27). **H** Representative bright field images of matrix contraction of pFs treated with serum EVs from HD, CRC liver MET, and CRC. (Shown: *n* = 3, Total: *n* = 12 per group). **I** Percentage of contraction shown in (**H**) of all samples calculated using imageJ (*n* = 12). Error bars depict mean ± SEM. P values were calculated by unpaired t-test except for (**B**) one-way ANOVA test was used. *p < 0.05, **p < 0.01, ***p < 0.001, ****p < 0.0001, ns not significant.

To determine the potential functional role of TIMP1 in ECM remodelling, CRC-EV-treated pFs were embedded in a 3D collagen Matrigel lattice [25], and the relative dimensional changes of the remodelled lattices were assessed at the initial time point of 0 h and after 48 h. CM- and serum-free medium (SFM)-treated pFs were used as controls. The percentage of changes in matrix area was significantly greater in CRC-EV-treated pFs (>35%) than in pFs treated with CM (<18%) or SFM (<5%) (Fig. 3A, B). To investigate whether TIMP1 levels in the treated pFs influence contraction ability, pFs were treated with EVs from TIMP1^WT^, TIMP1^OE^, and TIMP1^KO^ HCT116 cell lines. Indeed, the contraction of matrices embedded with pFs treated with TIMP1^OE^ EVs (50%) increased significantly as compared with TIMP1^WT^ (18%) or TIMP1^KO^ EV-treated pFs (3%) (Fig. 3C, D).

### Serum-derived TIMP1^EV^ from CRC patients promotes ECM remodelling

Recent studies have shown that the horizontal transfer of malignant traits from the primary tumour to distant organs is mediated by active EVs that are released into the blood circulation [26]. This prompted us to investigate if serum-derived EVs from cancer sera can recapitulate the upregulation of TIMP1 in recipient fibroblasts, promoting the ECM remodelling as a perquisite for the formation of a pre-metastatic niche. Therefore, pFs were treated with serum EVs from healthy donors (HD) (*n* = 20), patients with CRC liver METs (*n* = 46), and patients with primary CRC (*n* = 16). We found that *TIMP1* mRNA levels were significantly increased in pFs treated with serum EVs from CRC liver MET relative to those in pFs treated with serum EVs from HD (Fig. 3E). Although not statistically significant (*p* = 0.0949), *TIMP1* mRNA expression was also elevated in pFs treated with serum EVs from CRC compared to that in pFs treated with serum EVs from HD (Fig. 3E). Immunoblot analysis of pFs treated with serum EVs from HD, CRC liver MET, and CRC (*n* = 27/group) further confirmed the qPCR data, given that significant upregulation of TIMP1 protein expression in fibroblasts treated with serum EVs from patients with disease (CRC liver MET and CRC) was observed relative to that in those treated with serum EVs from HD (Figs. 3F, G and S5A). These results suggest that CRC cells release active TIMP1^EVs^ into the serum of patients with CRC, inducing TIMP1 upregulation in liver pFs.

As TIMP1 levels were significantly upregulated in pFs treated with serum-derived EVs from CRC patients, we investigated whether serum TIMP1^EV^-treated pFs can also induce ECM remodelling. Using our 3D ECM-remodelling assay, we measured the contracting ability of pFs treated with serum EVs from HD, CRC liver MET, and CRC (*n* = 12/group). We observed significant contraction of all collagen matrices embedded with pFs treated with serum EVs from CRC liver MET (22.16%) and CRC (21.73%) as compared with that observed following treatment with serum EVs from HD (3.24%) (Figs. 3H, I and S5B). To determine whether the differences in the ECM remodelling observed were linked to TIMP1^EV^-regulated TIMP1 expression, the EVs were pre-treated with a neutralising anti-TIMP1 antibody before pF treatment. The contraction of the collagen matrices was significantly abrogated in the presence of TIMP1 neutralising antibody compared to that of untreated controls (Fig. S5C, D). These results indicated that serum-derived TIMP1^EV^ from patients with CRC could promote ECM remodelling by regulating TIMP1 levels in pFs.

### TIMP1^EV^ is a non-invasive independent prognostic marker in colorectal liver metastases

Elevated TIMP1 levels in bio-fluids (serum and plasma) are often associated with poor prognosis in various cancers [27–30]. In accordance with our cell line model, we assessed TIMP1 protein levels in serum and their corresponding EVs in patients with advanced CRC (i.e., CRC liver MET) and patients with CRC having local primary disease at the time of blood collection (CRC); healthy donors with no known disease conditions (HD) were used as a control group (*n* = 20/group). Characterisation of isolated serum-derived EVs using NTA revealed the mean size (range: 150–200 nm), and TEM images confirmed cup-shaped EVs that expressed exosomal markers, including CD63, CD9, CD81, and TSG101 (Fig. S6A–C).

**Fig. 4:**
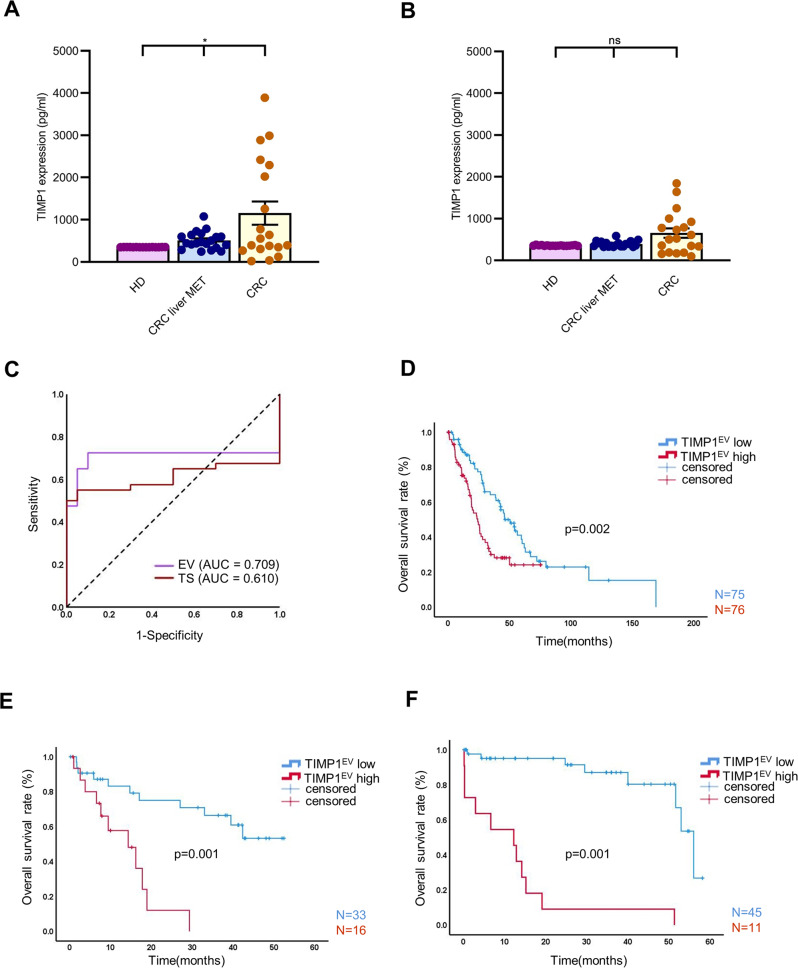
TIMP1^EV^ predicts poor clinical outcomes in patients with CRC liver MET. **A** TIMP1 abundance in serum EVs from healthy donors (HD), CRC liver MET and CRC detected by ELISA (*n* = 20 per group). **B** TIMP1 abundance in the corresponding serum from healthy donors (HD), CRC liver MET and CRC detected by ELISA (*n* = 20 per group). **C** Receiver operating characteristic (ROC) curves showing superior stratification power of TIMP1^EV^ (AUC = 709) compared to total serum TIMP1 levels (AUC = 0.610). **D** Kaplan-Meier curve of overall survival of patients with high or low TIMP1^EV^ based on the estimated median TIMP1^EV^ value in the discovery cohort. **E**, **F** Kaplan-Meier curve of overall survival of patients with high or low TIMP1^EV^ in validation cohort I (**E**) and Validation cohort II (**F**) based on the cut-off value of the TIMP1^EV^ estimated from the discovery cohort. P values were calculated by one-way ANOVA test (**A**, **B**) and log-rank test (**D**, **E**, and **F**). Error bars depict mean ± SEM. *p < 0.05, ns not significant.

First, we determined TIMP1 abundance in total serum and the corresponding EVs using ELISA, revealing a significantly higher TIMP1 abundance in the serum EVs of patients with CRC and CRC liver MET than in those of the HD group (Fig. 3A). However, no significant difference (*p* = 0.2270) in TIMP1 abundance in total serum was noted between patients with CRC or CRC liver MET and the HD group (Fig. 3B). A receiver operating characteristic curve analysis revealed that compared to soluble-TIMP1 from total serum, EV-associated TIMP1 was a superior diagnostic marker to distinguish between HD and patients with CRC (Fig. 4C) (EV-associated TIMP1: area under the curve (AUC): 0.709, soluble-TIMP1 from total serum: AUC: 0.61).

To further explore the relationship between TIMP1^EV^ levels and its clinical prognosis in patients with CRC liver MET, we evaluated the prognostic value of TIMP1^EV^ in patients with colorectal liver metastases from three independent cohorts. In the discovery cohort (*n* = 151) TIMP1^EV^ levels were quantified using ELISA. Patients were classified into TIMP1^EV^ high or TIMP1^EV^ low based on the median of TIMP1^EV^ ELISA values (TIMP1^EV^ = 1537 pg/ml). Univariate analysis revealed that a high expression of TIMP1^EV^ was associated with a shortened overall survival (OS) (TIMP1^EV^ high: median OS: 24.24 months, TIMP1^EV^ low: median OS: 50.50 months, *p* = 0.002) (Fig. 4D). Subsequently, we validated the cut-off value of 1537 pg/ml of TIMP1^EV^ in two independent cohorts (validation cohort I, *n* = 49 patients; validation cohort II, *n* = 56 patients). In both cohorts, a TIMP1^EV^ cut-off value > 1537 pg/ml was associated with a dismal OS (validation cohort I: TIMP1^EV^ high: median OS: 14.3 months, TIMP1^EV^ low: median OS non reached, mean OS: 37.98 months, *p* < 0.001; validation cohort II: TIMP1^EV^ high: median OS: 56.00 months, TIMP1^EV^ low: median OS non reached, mean OS: 12.26 months, *p* < 0.001) (Fig. 4E, F). To exclude sex bias in the prognostic significance of TIMP1^EV^, all three cohorts were reassessed based on sex. Kaplan-Meier analysis revealed no significant difference in the prognostic value of TIMP1^EV^ both in men and women (Table S1 and Fig. S7A-F). A multivariate analysis with the Cox proportional hazards regression model was performed to identify independent prognostic markers for OS. In all three cohorts, high TIMP1^EV^ remained the only significant factor for prognostic preoperative molecular stratification in patients with colorectal liver metastases (Table 1). Similarly, the multivariate analysis revealed no sex-specific impact on the OS (Table 1).

**Table 1:** Multivariable Cox Proportional Hazards Regression for CRC liver MET patients in the analysed cohorts.

Factor	Discovery Cohort				Validation Cohort I				Validation Cohort II (external)			
		95% C.I for Exp(B)				95% C.I for Exp(B)				95% C.I for Exp(B)		
	Exp(B)	Lower	Upper	*p* value	Exp(B)	Lower	Upper	*p* value	Exp(B)	Lower	Upper	*p* value
**Age**	1.015	0.991	1.039	0.220	1.010	0.968	1.055	0.635	0.988	0.945	1.034	0.612
**Sex**	1.108	0.687	1.787	0.673	1.361	0.498	3.722	0.548	1.046	0.369	2.963	0.933
**UICC**	0.993	0.979	1.008	0.362	1.076	0.653	1.773	0.774	100458.851	0.000	N/A	0.990
**KRAS**	1.001	0.995	1.006	0.774	2.906	0.625	13.503	0.174	N/A	N/A	N/A	N/A
**Pre-operative Theraphy**	1.515	0.908	2.529	0.112	0.995	0.985	1.006	0.372	N/A	N/A	N/A	N/A
**Median TIMP1**	1.902	1.218	2.968	**0.005**	5.694	1.952	16.613	**0.001**	18.250	5.588	59.606	**0.000**

EXP(B): Odds ratio; *CI* Confidence interval.

*P* values indicated in bold denote statistical significance.

### TIMP1^EV^ binds to HSP90AA

We then determined the molecular regulators of the TIMP1^EV^-mediated ECM-remodelling phenotype. First, we focused on identifying direct interacting partners of TIMP1, given that protein–protein interactions are a prerequisite for the activation of most signalling pathways. CD63 is the only known membrane receptor that directly interacts with TIMP1 in numerous malignancies, including hepatocellular carcinoma and other inflammatory diseases [31–34]. Previous studies identified heat-shock proteins (HSPs) as alternative potential candidates, with HSP90AA, a member of the HSP family, reportedly playing a pivotal role in fibroblast activation and the regulation of various client proteins, especially TIMP1, in fibrotic lung diseases [35].

**Fig. 5:**
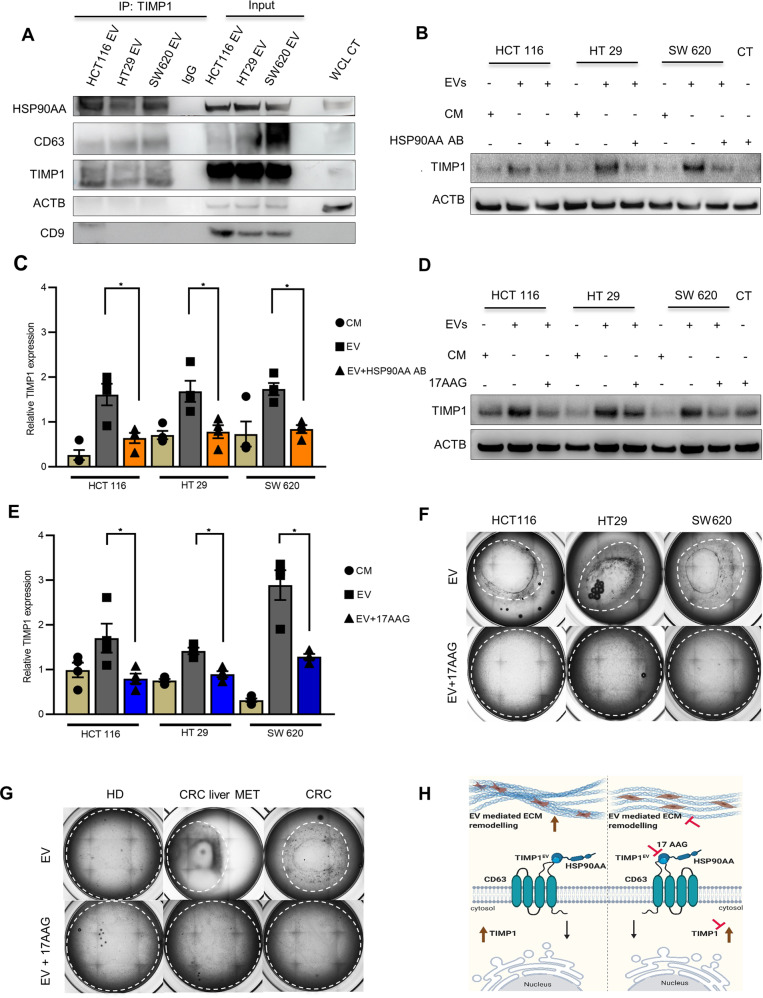
Inhibition of HSP90 attenuates TIMP1^EV^ mediated ECM remodelling. **A** Immunoblot of CD63, HSP90AA and TIMP1 following immunoprecipitation of TIMP1 from CRC-EVs (HCT116, HT29, and SW620) or Rabbit IgG as negative control. Input of lysates of HCT116, HT29, and SW620 EV protein were used as internal control. ACTB and CD9 were used as loading controls. Whole cell lysate (WCL) of SW620 EV treated pFs was used as an additional control. **B** Immunoblot analysis of TIMP1 expression in pFs treated with EVs and CM from HCT116, HT29 and SW620 in the presence of HSP90AA AB (20 ng/ml) and corresponding controls after 48 h. ACTB was used as loading control. **C** Densitometry quantification of TIMP1 expression normalised to ACTB shown in (**B**) using imageJ. **D** Immunoblot analysis of TIMP1 expression in pFs treated with EVs from HCT116, HT29 and SW620 in the presence or absence of 17AAG (0.3 µM) after 48 h. **E** Densitometry quantification of TIMP1 expression normalized to ACTB shown in (**D**) using imageJ. **F** Representative bright field images of collagen matrigel lattices with embedded pFs treated with CRC-EVs (HCT116, HT29 and SW620) in the presence or absence of 17AAG (0.3 µM) after 48 h. **G** Representative bright field images of collagen matrigel lattices with embedded pFs treated with serum EVs from HD, CRC liver MET, and CRC treated pFs in the presence or absence of 17AAG (0.3 µM). **H** Graphical overview of the TIMP1^EV^ mediated ECM remodelling. TIMP1^EV^ binds to HSP90AA and CD63 receptor on the cell surface of fibroblasts to regulate TIMP1 in the recipient cells leading to ECM remodelling (left). Inhibition of HSP90 or TIMP1^EV^ levels affects TIMP1 mediated ECM remodelling (right). All experiments were performed at least 3 times. Error bars depict mean ± SEM. P values were calculated by unpaired t test. *p < 0.05.

To examine putative molecular interactions between TIMP1^EV^, HSP90AA, and CD63, we performed immunoprecipitation of TIMP1 from EVs of CRC cell lines, with rabbit isotype control IgG used as a negative control. Western blot analysis of the isolated immunocomplexes showed that the anti-TIMP1 antibody co-immunoprecipitated HSP90AA but not CD63, suggesting that HSP90AA is bound to TIMP1^EV^ and does not interact with CD63 (Fig. 5A).

As a reciprocal experiment, we investigated whether TIMP1 interacts with HSP90AA and CD63 in pFs conditioned with CRC-EVs. Following treatment of pFs with CRC-EVs (HCT116, HT29, and SW620) for 48 h prior to TIMP1 immunoprecipitation, immunoblot analysis of the isolated immunocomplexes showed a significant increase in co-immunoprecipitation of CD63 and HSP90AA upon CRC-EV treatment. In CM-treated pFs (used as controls), neither HSP90AA nor CD63 were co-immunoprecipitated with TIMP1 (Fig. S8A). These results showed that HSP90AA is constitutively bound to TIMP1^EV^ and might interact with the CD63 receptor of recipient fibroblasts to promote downstream signalling functions.

### HSP90 inhibition interferes with TIMP1 protein stabilisation

There are currently no known inhibitors of TIMP1 and CD63. As the immunoprecipitation data indicated that HSP90AA constitutively binds to TIMP1^EV^, we hypothesised that HSP90AA might be important for TIMP1 stabilisation and is a potential target candidate for its inhibition. To elucidate the potential outcome of HSP90AA-mediated inhibition, we devised two-way approaches to inhibit the action of HSP90AA. In the first approach, CRC-EVs were pre-treated with an anti-HSP90AA antibody for 2 h at 37 °C, followed by fibroblast treatment in the presence of an anti-HSP90AA antibody (Fig. 5B). In the second approach, pFs were treated with CRC-EVs along with 17AAG (a HSP90AA inhibitor) (Fig. 5D) at a dose optimised according to previous studies [35]. Immunoblot analysis revealed a significant attenuation of TIMP1 upregulation in both approaches as compared with corresponding controls (Fig. 5B–E), suggesting HSP90AA as a potential target for inhibiting TIMP1 upregulation in TIMP1^EV^-treated pFs.

### 17AAG attenuates TIMP1^EV^-mediated ECM remodelling

We then determined whether attenuation of cellular TIMP1 level through HSP90AA inhibition can impede the ECM-remodelling phenotype mediated by TIMP1^EV^. Using our 3D ECM-remodelling assay model, we evaluated the contracting ability of collagen lattices embedded with pFs treated with EVs from a CRC cell line in the presence and absence of 17AAG. Remarkably, in the presence of 17AAG, the contraction was significantly abrogated in collagen lattices embedded with CRC-EV-treated pFs relative to those in CM- and SFM-treated controls (Figs. 5F and S8B). Similarly, we analysed the contracting ability of collagen lattices embedded with pFs treated with serum EVs from HD, CRC liver MET, and CRC (*n* = 9/group) in the presence or absence of 17AAG. In agreement with the results of our cell line-derived EV assays, pFs treated with serum-derived EVs from patients with disease (CRC liver MET and CRC) showed a complete abrogation of contraction of the collagen lattices in the presence of 17AAG as compared with untreated controls (Figs. 5G and S8C). Interestingly, this abrogation was comparable to the degree of contraction observed in collagen lattices embedded with HD-derived serum-EV-treated pFs, irrespective of the addition of 17AAG (Fig. 5G). The effect of 17AAG on TIMP1^EV^-mediated ECM remodelling was verified using pFs treated with EVs from TIMP1KO cell line in the presence and absence of 17AAG. No significant change in contraction was observed in both conditions (Fig. S8D, E). Taken together, these results suggest that inhibition of HSP90AA attenuates TIMP1^EV^-mediated ECM remodelling, rendering TIMP1-EV a potential therapeutic target.

## Discussion

The intracellular transport of EVs is believed to be an efficient means of modulating cell signalling and biological functions in recipient cells, and tumour-derived EVs promote metastasis by conditioning stromal cells [36]. In the present study, we focused on establishing a model system enabling the investigation of the effect of CRC-EV-conditioned pFs. In the first approach, we validated TIMP1, a two-domain protein harbouring metalloproteinase-inhibitory functions, as being specifically elevated in stromal fibroblasts at the invasive front of CRC liver MET. In accordance with our hypothesis that the upregulation of TIMP1 level in stromal fibroblasts adjacent to tumour cells is mediated by an EV-mediated crosstalk, we showed that TIMP1 was selectively enriched in CRC-EVs and that TIMP1^EV^ regulated TIMP1 levels in recipient fibroblasts. These data are consistent with the findings of a previous study demonstrating that high TIMP1 levels stimulate the accumulation of cancer-associated fibroblasts through pro-tumorigenic gene regulation of fibroblasts [37].

Although interest in TIMP1 has increased based on its role as a protein biomarker in various cancers according to findings in solid or liquid biopsies, its precise functional role as a tumour promotor has remained unclear. This study identified the role of TIMP1^EV^ as a modulator of the ECM. Our 3D ECM remodelling assays results suggest that elevated TIMP1 levels affect cell dynamics in fibroblasts, which then induce matrix remodelling, subsequently resulting in altered mechanical properties of the tumour microenvironment. This was further corroborated in a study by Dechene et al. [38] revealing that patients with continuously increasing TIMP1 levels exhibited liver stiffness during acute liver failure [38]. Another study noted that increased matrix rigidity in hepatic stellate cells leads to an increased secretion of TIMP1 into the extracellular space and decreased intracellular TIMP1 levels, thereby perpetuating fibrosis [39]. This mechanism could be attributed to our observations of low cellular TIMP1 in CRC cell lines as compared with those in TIMP1^EV^; the secretion of TIMP1-enriched EVs into the extracellular space could represent a process employed by cancer cells to decrease their stiffness and accelerate migration. In line with previous reports [26], we can show that this activation and remodelling of the ECM is not only relevant in *vitro*, but that serum-derived EVs from cancer patients can transfer those malignant traits to recipient fibroblasts. Accordingly, we showed that serum TIMP1^EV^ from CRC patients induced TIMP1 upregulation in recipient liver fibroblasts. Serum TIMP1^EV^-mediated TIMP1 upregulation in fibroblasts might underlie the changes in matrix remodelling observed using our 3D ECM-remodelling assay. Since a remodelled ECM is considered an important aspect of the “soil effect”, serum TIMP1^EV^-mediated ECM remodelling exerted by the pFs could be a putative precursor to the establishment of a metastatic niche in the liver to support tumour-cell colonisation. This interpretation is supported by Seubert et al. [5] who have demonstrated that elevated systemic TIMP1 level creates a pre-metastatic niche in the liver. In the context of a critical discussion, however, we have not differentiated in more detail whether TIMP1^EV^-treated fibroblasts transform into myofibroblasts. This point might be addressed in follow-up studies investigating the phenotype-changing potential of TIMP1^EV^ for the transformation of fibroblast into myofibroblasts.

Moreover, in accordance with the REMARK guidelines [40], we demonstrated that EV-bound TIMP1 is a robust circulating biomarker for a non-invasive, preoperative risk stratification in patients with colorectal liver metastases. Liver resections in particular are associated with a high risk of postoperative morbidity and mortality. Therefore, a preoperative risk assessment for long-term oncological outcomes might be highly relevant. In this context, determining preoperative EV-bound TIMP1 could be an additional decision aid for molecular risk stratification and personalised therapy guidance. Aditionally, our data indicate that compared to the analysis of soluble TIMP1 from total serum, the determination of EV-associated TIMP1 is superior for non-invasive diagnoses. Although EV-associated TIMP1 is not a perfect diagnostic marker, the AUC was greater than the AUC of soluble serum TIMP1. In fact, these data support the assumption that the enrichment of EVs from total serum can increase the yield of tumour-specific protein markers. Conclusively, this EV-enrichment may help to minimise false-positive results in healthy individuals and increase the diagnostic power of liquid biopsy-associated markers.

Finally, we investigated whether EV-bound TIMP1 is a potential therapeutic target for the treatment of CRC. The extracellular molecular chaperone HSP90 reportedly stabilises matrix metalloproteinase 2, which promotes tumour-cell invasion [41]. Here, we showed that HSP90AA is constitutively bound to TIMP1^EV^ and that targeting HSP90AA leads to TIMP1 downregulation. Furthermore, we demonstrated that targeting TIMP1 impaired ECM remodelling. We subsequently showed that 17AAG, a HSP90 inhibitor, significantly abrogated TIMP1 upregulation and inhibited ECM remodelling, thereby suggesting it as a potential drug target. This finding was supported in a previous study showing that HSP90 supports fibroblast activation and ECM production and validating HSP90 inhibitors as potential therapeutic candidates for the treatment of fibrotic lung disease [35]. Additionally, this previous study identified TIMP1 as a target protein in the ECM, whose level is downregulated upon HSP90 inhibition. Targeting HSP90 represents a promising strategy for anticancer drug development [42]. Therefore, further studies might focus on establishing 17AAG and heparin as potential candidates for combination therapy to curtail the priming of secondary organs and furthering of metastasis in patients with CRC.

In conclusion, these data provide novel insights into the onset of ECM remodelling exerted by liver stromal cells being initiated by the uptake of TIMP1^EV^; these findings offer novel opportunities to study TIMP1^EV^ as a potential candidate for prognostic stratification and therapeutic intervention to reduce the prospect of liver metastasis from CRC.

## Materials and methods

### 3D ECM-remodelling assay

Fibroblasts are sensitive to ECM mechanics. To analyse the ECM-remodelling ability of TIMP1^EV^-treated fibroblasts, gel mixtures containing collagen type-I (high concentration, rat tail; #354349; stock concentration 9.33 mg/ml; Corning, Corning, NY, USA), and Matrigel matrix basement membrane (#354230; stock concentration 9.7 mg/ml; Corning) were prepared with 10% 10 × Dulbecco’s modified Eagle medium (Sigma-Aldrich, St. Louis, MO, USA) and 10% foetal bovine serum to yield a final gel concentration of 4 mg/ml Collagen type-I and 2 mg/ml Matrigel. The gel mixture was neutralised with 1 M NaOH (Sigma-Aldrich), and primary liver fibroblasts (pF; 2.5 × 10^6^) pre-treated with cell line or serum-derived EVs, and corresponding controls for 6 h were added to the gel mixture. Thereafter, 200 µl of the gel-cell mixture was added to the wells of a 48-well plate that was pre-treated with 3% bovine serum albumin (Sigma-Aldrich) incubation for 1 h, followed by washing with phosphate-buffered saline (Sigma-Aldrich) and air drying for 10 min. Gels were set at 37 °C for 1 h and incubated with culture media with or without EVs for 48 h at 37 °C, with media changes every 24 h. The ECM remodelling assay was also performed using EVs pre-treated with TIMP1 and HSP90AA neutralising antibodies (see Supplementary Table S4). In HSP90 inhibition assays, the HSP90AA antibody and 17AAG were present in the culture media throughout the assay.

For further details regarding the materials and methods used, please refer to the Supplementary Data.

## Supplementary information

Supplementary Data

Supplementary Table 1-4
