# Supplementary material for: Extravesicular TIMP-1 is a non-invasive independent prognostic marker and potential therapeutic target in colorectal liver metastases
Source: Oncogene. 2022 Feb 9;41(12):1809–20. doi: 10.1038/s41388-022-02218-9 (PMC8933275; doi:10.1038/s41388-022-02218-9)
Supplement: Supplementary file 1 — Supplementary Data [file 41388_2022_2218_MOESM1_ESM.docx]

**Extravesicular TIMP-1 is a non-invasive independent prognostic marker and potential therapeutic target in colorectal liver metastases**

Venkatesh Sadananda Rao, Qianyu Gu, Sandra Tzschentke, Kuailu Lin, Nicole Ziegler, May-Linn Thepkaysone, Fang Cheng Wong, Heike Polster, Nathalie Buck, Carina Riediger, Jonas Weiße, Tony Gutschner, Susanne Michen, Achim Temme, Lena Seifert, Adrian M Seifert, Martin Schneider, Franziska Baenke, Jürgen Weitz, Christoph Kahlert

Table of contents

Supplementary materials and methods………………………………………………………...2

Suppplementary references…………………………………………………………………….9

Supplementary Figure S1……………………………………………………………………..10

Supplementary Figure S2……………………………………………………………………..11

Supplementary Figure S3……………………………………………………………………..12

Supplementary Figure S4……………………………………………………………………..13

Supplementary Figure S5……………………………………………………………………..14

Supplementary Figure S6……………………………………………………………………..15

Supplementary Figure S7……………………………………………………………………..16

Supplementary Figure S8……………………………………………………………………..17

Original data…………………………………………………………………………………..18

**Patients**

Paraffin-embedded samples of primary colorectal adenocarcinomas (CRC) and CRC liver metastases (CRC liver MET) were included from 81 and 80 patients, respectively, undergoing curative surgery between 2011 and 2015 at the Department of Visceral, Thoracic and Vascular surgery, at the University Hospital Carl Gustav Carus, Dresden. The median follow-up time for the patients with primary CRC was 44 months and that for patients with CRC liver MET was 51 months. A written informed consent regarding tissue sampling was obtained from all patients. The study protocol was approved by the ethics committee of Dresden (EK76032013). Further details regarding the patient samples analysed can be found in Supplementary Table S2.

**Immunohistochemistry**

Primary CRC and liver tissue with CRC metastasis were surgically excised, assessed by pathologists and embedded into paraffin at the Department of Pathology, University Hospital Carl Gustav Carus Dresden. Tissue sections (8 µm) were cut using a microtome (Leica biosystems, Wetzlar, Germany) and placed on glass slides (Superfrost® Plus, ThermoScientific). Following primary antibodies were used: TIMP1 D10E6 and rabbit (DA1E) mAb IgG XP® isotype control from Cell signalling (Danvers, MA, USA). The Signalstain® Boost IHC/HRP rabbit (Cell Signaling Technology, Danvers, MA, USA) was used as secondary antibody. Stained sections were imaged and scanned by Panoramic SCAN (3D Histech, Budapest, Hungary). The slides were evaluated blindly by a board-certified surgeon and an independent researcher and a score was given for each sample. The staining intensity of TIMP1 was classified as 0 (negative), 1 (weak), 2 (moderate) or 3 (strong).

**Cell lines**

Human colorectal carcinoma (HCT-116 (ATCC CCL-247), HT-29 (ATCC HTB-38), SW620 (ATCC CCL-227)) and human skin fibroblast (BJ (ATCC CRL-2522)) cell lines were purchased from American Type Culture Collection (ATCC, Rockville, CT, USA). All cell lines were cultured in Dulbecco’s modified eagle medium (DMEM, Gibco®, Thermo Fisher Scientific, MA, USA) supplemented with 10% fetal bovine serum (FBS, Gibco®, Thermo Fisher Scientific) and 1% penicillin/streptomycin (Biochrom GbmH, Berlin, Germany). The cells were incubated at 37 °C in a humidified 5% CO2 incubator and subcultured by trypsin-EDTA treatment when they achieved ~80% confluency. The cells were tested regularly for mycoplasma and their STR profile was routinely checked.

**Primary liver fibroblast cell lines**

Primary liver fibroblasts (pF) were established from excised liver sections (non-cancerous region) of patients undergoing liver resection surgery at the Department of Visceral, Thoracic and Vascular Surgery at the University Hospital Carl Gustav Carus Dresden. All subjects gave written informed consent. The study was approved by the Ethics Committee Dresden (approval number EK76032013).

The liver tissue was washed intensely with 1% Antibiotic-Antimycotic (Gibco®, Thermo Fisher Scientific, Waltham, MA, USA) in DPBS (Gibco®, Thermo Fisher Scientific) for 60 min on a rocker with frequent change of the wash buffer. The clean tissue was then minced into pieces of 1 x 1 mm in size, distributed on 6-well plates and dried for 30 min until the tissue pieces adhered to the plate. Fibroblast culture medium (Dulbecco’s modified eagle medium (DMEM, Gibco®, Thermo Fisher Scientific) supplemented with 20% FCS and 1% Anti-Anti) and keratinocyte serum free medium (KSFM, Gibco®, Thermo Fisher Scientific) including the provided supplements, and 0.1% Primocin (InvivoGen, San Diego, CA, USA) at a 2:1 ratio was then added to the plate and incubated at 37°C, 5% CO_2_. In 2-4 weeks, the sprouted fibroblasts from the tissue pieces were expanded and cultured in fibroblast culture medium.

**EV Isolation from CRC cell lines**

The CRC cell lines were cultured in 875 m² multiflasks (Falcon®, Corning®, Corning, NY, USA). Cells reaching a density of 90‑95 % were washed with Dulbecco’s phosphate buffered saline (PBS: Gibco®, Thermo Fisher Scientific) prior to addition of 125 ml starvation medium (DMEM only). After 48 h, the conditioned medium (CM) was harvested and subjected to differential centrifugation. Briefly, the CM was centrifuged at 2000 x g for 30 min at 4 °C, followed by filtration using a 0.2 µm PES filter (corning). The flow through was then subjected to overnight ultracentrifugation at 100,000 x g at 4 °C. Based on the downstream analysis, the EV pellet was processed for Transmission electron microscopy (TEM), by re-suspending in PBS, for protein analysis, using RIPA buffer including 1x Halt™ Protease- and Phosphatase-Inhibitor-Cocktail or for functional analyses, re-suspending in starvation medium.

**Isolation of serum-derived EVs**

Blood samples were obtained from patients undergoing surgery at the Department of Visceral, Thoracic and Vascular Surgery at the University Hospital Carl Gustav Carus Dresden immediately before surgery. Venous blood samples were collected in EDTA containing monovettes (Sarstedt, Nürmbrecht, Germany) and processed within 4 hours. The samples were centrifuged at 1,500 x g for 12 min at 4°C and retrieved serum was stored at -80 °C. The discovery cohort and validation cohort I included serum samples from colorectal liver metastases who underwent curative resection at the Department of Surgery, Dresden University Hospital between 2009 and 2017.The validation cohort II is an external validation cohort that included pre-surgical serum samples of patients with colorectal liver metastases collected from University Hospital Heidelberg between 2012 and 2015. Further details regarding the patient samples analysed can be found in Supplementary Table S3.

For EV isolation, 1ml serum was thawed on ice and transferred to a 1.5ml Eppendorf tube to perform the EV isolation using Exo-spin^TM^ exosome purification kit (EX01-50: CELL guidance systems, Cambridge, UK) according to the manufacturer’s protocol (human breast milk protocol).

**Characterisation of EVs**

Isolated EVs were assessed by nanoparticle tracking analysis (NTA) using ZetaView® (Particle Metrix, Inning am Ammersee, Germany) and transmission electron microscopy (TEM) imaging. For TEM, EVs were adsorbed onto 400-mesh carbon/formvar grids, rinsed with PBS and ddH2O, air dried, and counterstained using uranyl acetate. Images were acquired using a Tecnai Bio-Twin transmission electron microscope (Field Electron and Ion Company (FEI), Hillsboro, OR, USA) with an AMT CCD Camera (Advanced Microscopy Techniques, Woburn, MA, USA). Immunoblot analyses were performed on EVs lysed with RIPA buffer (150 mM NaCl, 1% Triton X 100, 0.5% SDS, 50 mM Tris) including 1× Halt™ Protease- and Phosphatase-Inhibitor-Cocktail (Thermo Fisher Scientific). Primary antibodies used for the detection of proteins of interest are listed in Supplementary Table S4.

**Treatment of Fibroblasts with EVs**

BJ and pF cells were seeded in a 6-well plate at a density of 2 × 10^5^ cells per well in DMEM supplemented with 10% FBS and 1% penicillin/streptomycin and incubated in a humidified 5% CO2 atmosphere for 24 h. Cells at 70–80% confluency were treated with CRC cell line- or serum-derived EVs at a concentration of 10^9^ EVs / well (5,000 particles / cell) for a period of 24 h and 48 h for real time quantitative polymerase chain reaction (RT-qPCR) and immunoblot experiments, respectively. EV treatment assays were performed on three pF cell lines established from three different donors.

**TIMP1 Knock-Out (KO) and Over-Expression (OE)**

HCT116 TIMP1^KO^ cells were generated by using Lipofectamine LTX/PLUS (Invitrogen, Waltham, MA, USA) and the PX330-hCas9-P2A-mCherry vector carrying guide RNAs (Supplementary Table S4) targeting human TIMP1. Single guide RNAs were chosen using Genetic Perturbation Platform sgRNA Designer from Broad Institute. Briefly, mcherry-expressing cells were single-cell-sorted into 96-well plates for clonal growth. TIMP1 depletion was evaluated by immunoblot.

For generation of TIMP1^OE^ HCT116 cells, the self-inactivating lentiviral pHATtrick vector [1] containing an internal spleen focus forming virus (SFFV) U3 promoter followed by a multiple cloning site, and a T2A Thosea asigna virus element fused in frame to a neomycin-resistance gene was used. A full-length chemically synthesized cDNA of TIMP1 fused with a c-myc and His6x epitope tag was obtained from Eurofins Genomics (Ebersberg, Germany) and cloned via the restriction sites AgeI and NotI in frame to T2A-neomycinR, resulting in pHATtrick-TIMP1-myc-His6x-NeoR. The vector was verified by DNA sequencing (Microsynth Seqlab, Goettingen, Germany).

Lentiviral particles cells were produced by a transient three vector packaging protocol [1]. 1 × 10^5^ HCT116 cells in a 6-well were transduced with 1 ml of lentiviral supernatant, 1 ml complete RPMI-1640 medium and 8 µg/ml polybrene (Sigma-Aldrich, St. Louis, MO, USA) for 24 h. The cells were subsequently selected with 400 µg/ml geneticin (G-418) (Life Technologies, Carlsbad, CA, USA) for 10 days. TIMP1 overexpression was confirmed by immunoblot.

**RNA isolation and RT-qPCR**

Total RNA from treated fibroblasts and EVs was extracted using miRNeasy (Qiagen, Hilden, Germany) according to manufacturer’s instructions. RNA quality and concentration were evaluated using Nanodrop (GE Healthcare, Chicago, IL, USA) and Qubit 4 (Invitrogen, Carlsbad, CA, USA). High Capacity cDNA Reverse Transcription Kit (Applied Biosystems, Foster City, CA, USA) was used to reverse transcribe total RNA extracted from cells. Quantitative PCR was performed to analyze the gene expression profile of listed genes using Step One plus System (Applied Biosystems). Gene list and primers sequences are listed in (Supplementary Table S4). Data were normalised using the ΔΔ cycle threshold (ΔΔCT) method using ACTB (β-actin) as a housekeeping gene. CM-treated fibroblasts were used as control.

**Immunoblotting and co-immunoprecipitation**

Total protein was isolated from treated fibroblasts and EVs using RIPA buffer with 1x Halt^TM^ inhibitor-cocktail (Roche, Basel, Switzerland) after washing 3 times with PBS. Protein samples were loaded onto Novex 4–12% Tris-glycine gels (Life Technologies, Carlsbad, CA, USA) and transferred onto nitrocellulose membranes. Proteins of interest were detected using primary and HRP-conjugated secondary antibodies (see Supplementary Table S4) and imaged by Immobilon Western HRP Substrat (Merck, Darmstadt, Germany). Blots were imaged at a G:Box Chemi XT4 imager (Syngene, Cambridge, UK) and Fusion Fx ( Vilber, Collegien, France). The ratio between the TIMP1 and ACTB band intensities of each sample was measured using ImageJ software (NIH, Bethesda, MD, USA) for the quantification of the TIMP1 abundance.

For co-immunoprecipitation studies, the treated pFs were harvested after indicated treatment, washed with PBS, and lysed in RIPA buffer with 1x Halt^TM^ inhibitor cocktail (Roche). Lysates were pre-cleared with protein A beads for 30 minutes followed by incubation with primary antibody (1 µg) overnight. The immunoprecipitated fraction was washed 3 times with lysis buffer, solubilised in SDS buffer, and equal amounts were loaded on SDS-PAGE gels.

**ELISA**

Human TIMP-1 ELISA Kit (RAB0467, Sigma-Aldrich, St. Louis, MO, USA) was used to evaluate TIMP1 abundance in total serum and corresponding EVs of healthy controls and CRC patients, according to the manufacturer’s instructions. Total protein content was normalized by BCA assay.

**Anti-TIMP1 antibody treatment**

Serum-derived EVs were pre-treated with anti-TIMP1 AB (1 µg) for 2 h at 37°C in a shaker followed by EV treatment of PFs for 6 h.

**17 AAG and HSP90AA antibody treatment**

To assess the effects of 17-AAG and HSP90AA antibody (AB) on TIMP1, pFs were seeded in a 6-well plate at a density of 2 × 10^5^ cells for 24h. Cells at 70–80% confluency were treated with CRC-derived EVs for 48 h in combination with HSP90AA AB (20 ng/ml), 17AAG (0.5µM) or treatment control. For HS90AA antibody treatment, the CRC EVs were pre-treated with HSP90AA AB for 2 h at 37°C in a shaker followed by EV treatment in the presence of the AB.

**Stimulation assay**

To assess the cytokine-like effects of TIMP1, pFs were seeded in a 6-well plate at a density of 2 × 10^5^ cells for 24h followed by serum starvation overnight. The cells were then stimulated with increasing concentration of human recombinant TIMP1 sourced from mouse myeloma cells (970-TM-010, R&D systems, MN, USA). At various time points, the treated cells were harvested and TIMP1 expression levels were determined by quantitative RT-PCR and Western Blot.

**Statistical analysis and reproducibility**

The experiments were performed as biological replicates and repeated independently. Statistical analyses for all the experiments were performed in GraphPad Prism (San Diego, CA, USA,Version 9.0.0) and IBM SPSS (NY, USA). Mann‑Whitney‑U test and Kruskal-Wallis test (one-way ANNOVA) were used. Error bars in graphs represent mean ± s.e.m with interquartile range. The number of biological replicates for each experiment and the sample size of each experimental group / condition are provided in the figure legends. To calculate differential TIMP1 expression and contractility (ECM assay) in fibroblasts upon serum-derived EV treatment, the area under the curve (AUC) was calculated through receiver operating characteristics (ROC) curves. Likewise, differences of EV-associated TIMP1 and soluble TIMP1 in human serum samples was assessed by calculating the area under the curve (AUC) by receiver operating characteristics (ROC) curves. The Kaplan-Meier method was used to construct survival curves that were compared using the log-rank test. A Cox proportional hazards regression analysis was used to assess independent predictors of overall survival. A p-value ≤ 0.05 was considered statistically significant. Adobe Photoshop 2021 and BioRender were used for image editing and illustrations.

**Data availability**

Source data for all figures are provided with the paper. Further details about the materials and all other data are available from the corresponding author upon reasonable request.

**Supplementary References**

1 Töpfer K, Cartellieri M, Michen S, Wiedemuth R, Müller N, Lindemann D *et al.* DAP12-Based Activating Chimeric Antigen Receptor for NK Cell Tumor Immunotherapy. *J Immunol* 2015. doi:10.4049/jimmunol.1400330.


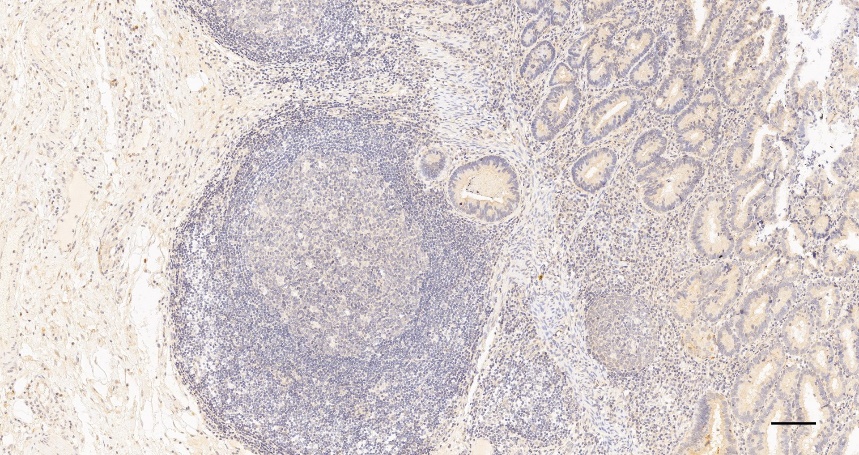

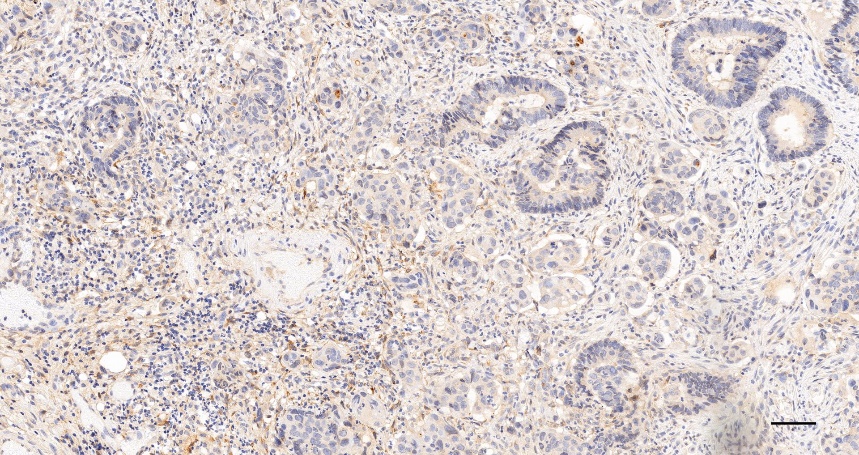

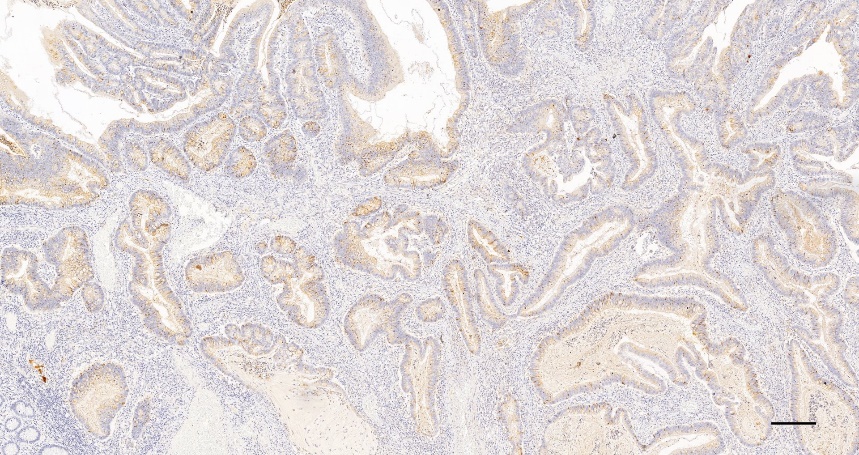

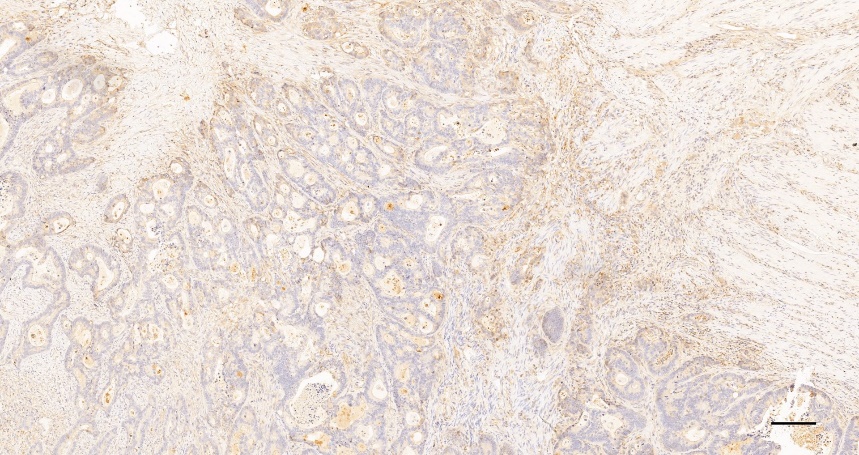

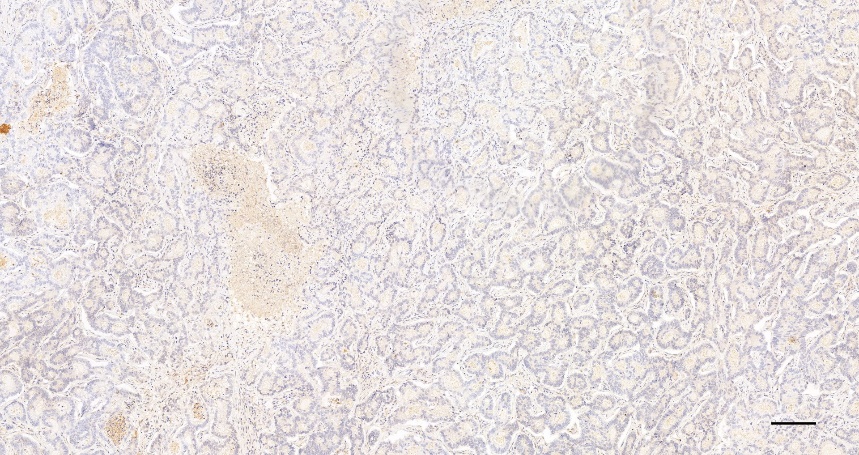

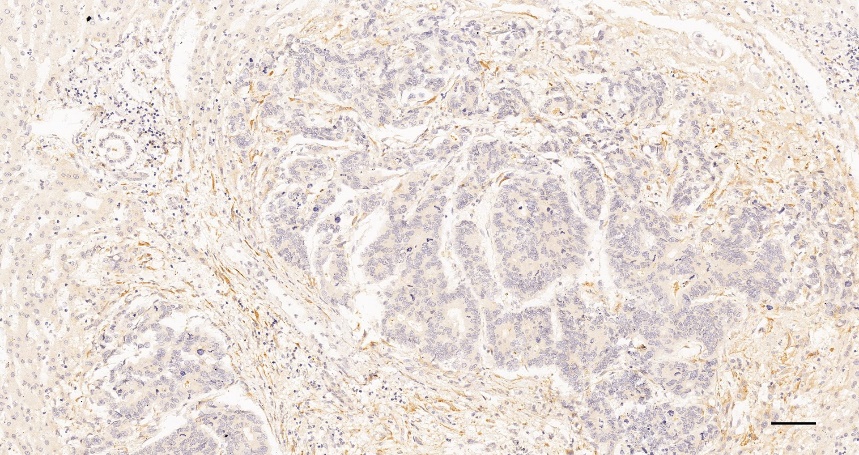

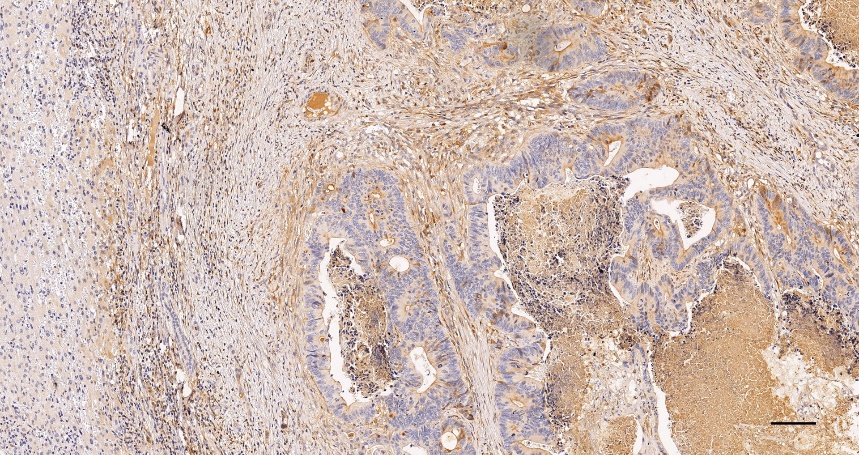

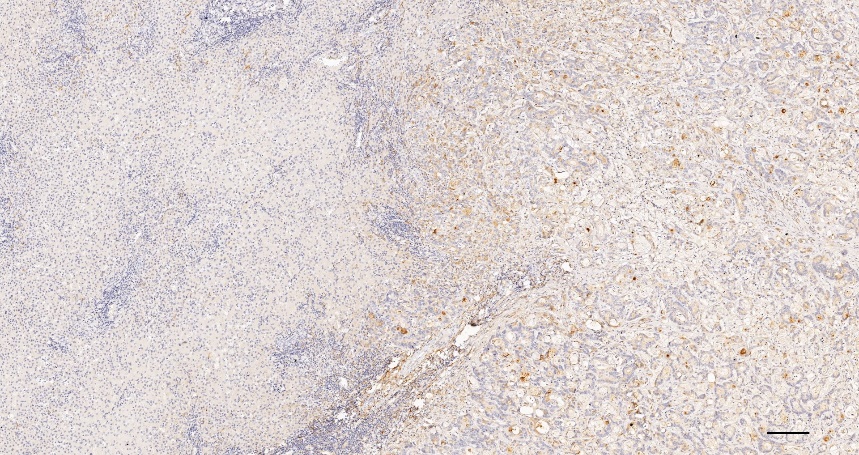


**Supplementary Fig.S1. Visual scoring system used to assess TIMP1 intensity in CRC and CRC liver MET tissues.** Scores 0-3 relate to intensity of TIMP1 staining in the IM and CT of Tumor and stromal region. 3-6 sections per tissue sample were assessed and the mean score plotted for each sample. Scale bar: 100μm.

CRC

CRC liver MET

Score 1

Score 2

Score 3

Score 0

pF 1 (male)

BJ (male)

CM

EV

CM

EV

CM

EV

HCT 116 (male)

HT 29 (female)

SW 620 (male)


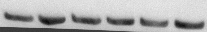

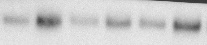


TIMP1

ACTB

ACTB

CM

EV

CM

EV

CM

EV

HCT 116 (male)

HT 29 (female)

SW 620 (male)


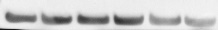

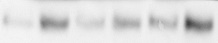


TIMP1

**Supplementary Fig.S2. Gender-based analysis of CRC-EV mediated TIMP1 upregulation.** Immunoblot analysis of TIMP1 expression in the fibroblasts (BJ-male, pF1-male, pF2- female, pF3-female) treated with EVs and CM from HCT116 (male), HT29 (female) and SW620 (male).

pF 3 (Female)

CM

EV

CM

EV

CM

EV

HCT 116 (male)

HT 29 (female)

SW 620 (male)


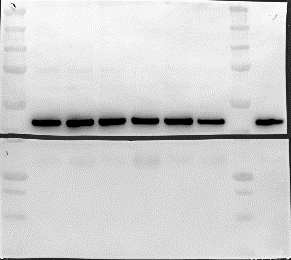

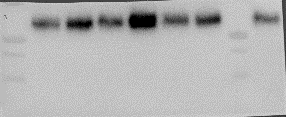


ACTB

CM

EV

CM

EV

CM

EV

HCT 116 (male)

HT 29 (female)

SW 620 (male)


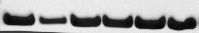

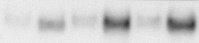


ACTB

TIMP1

TIMP1

pF 2 (Female)


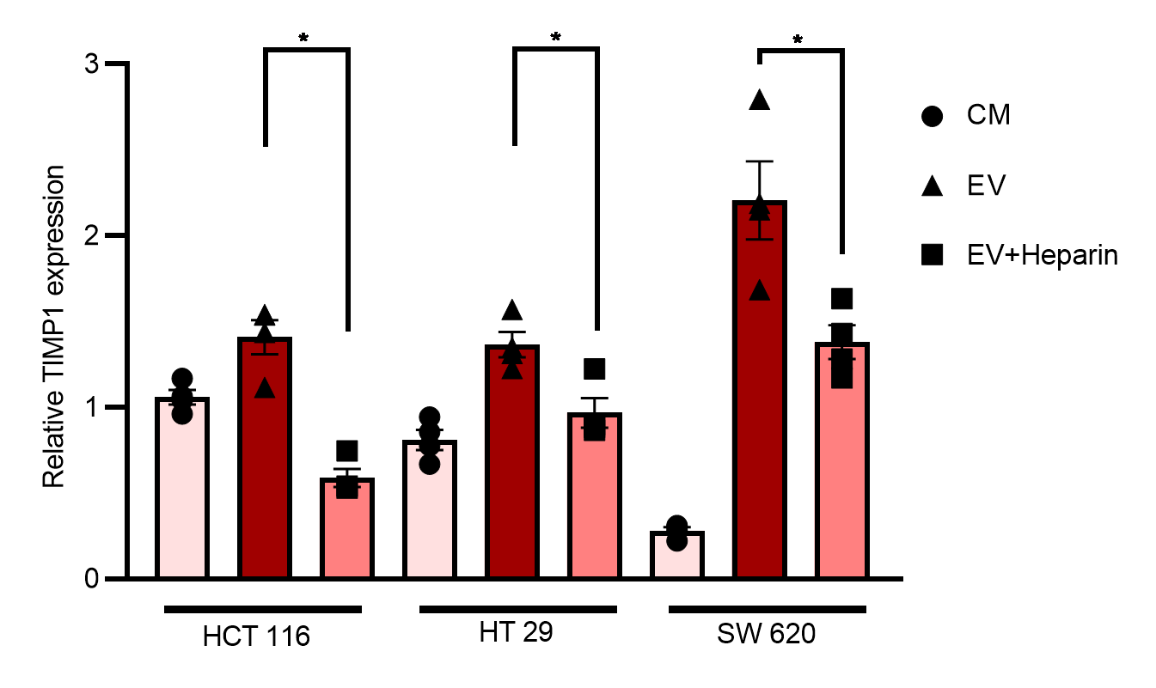

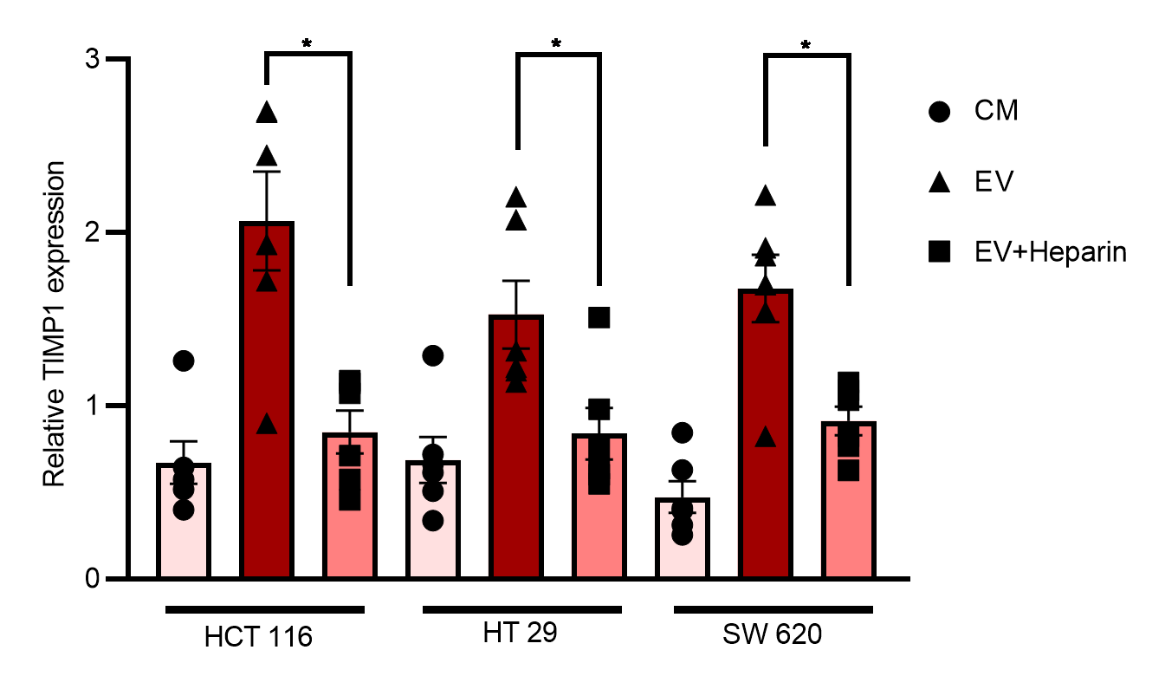

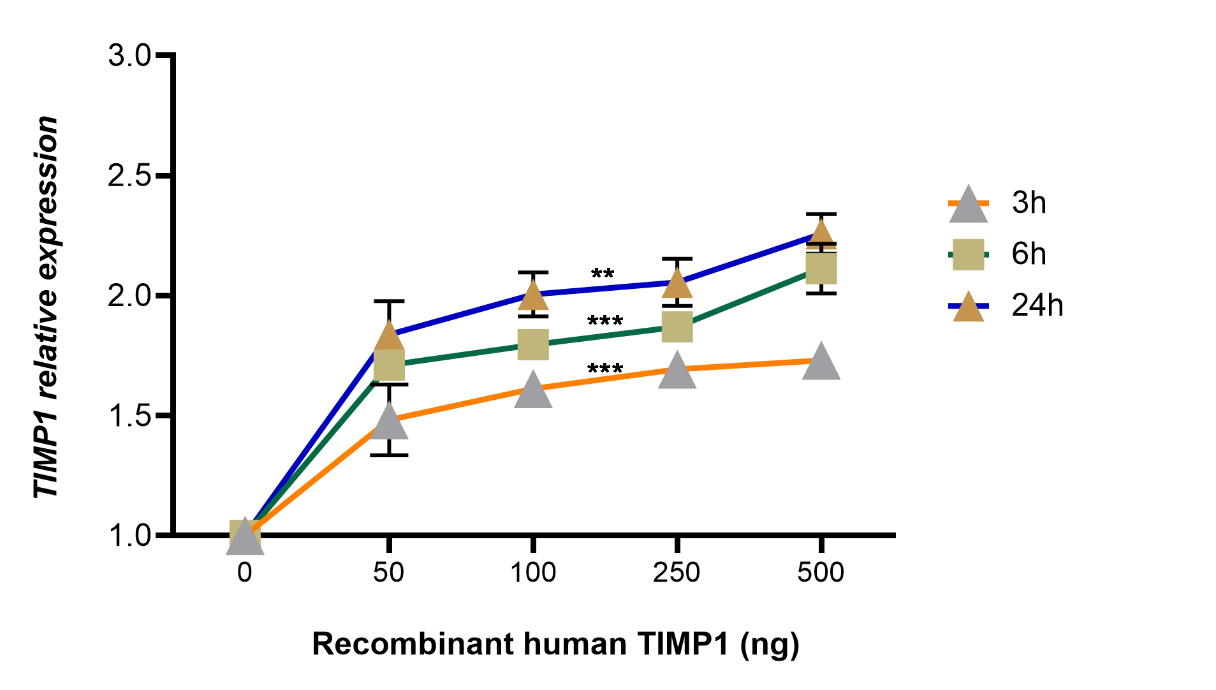

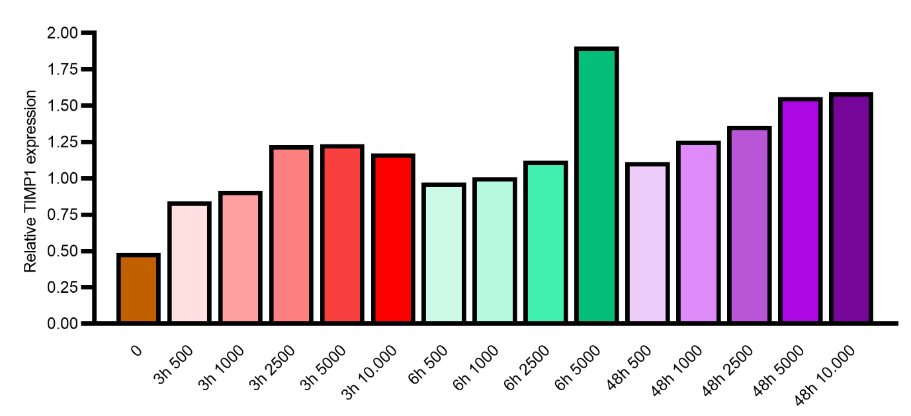


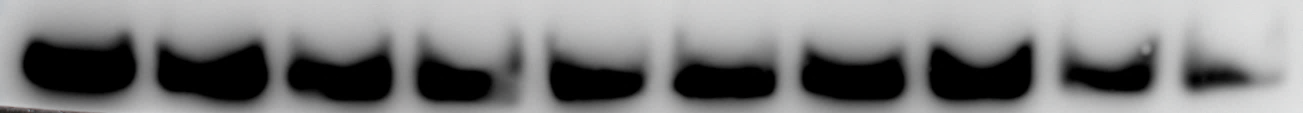

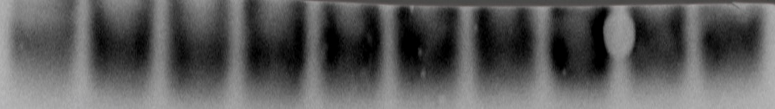


0

500

Recombinant TIMP1 (ng)

1000

2500

5000

10000

500

1000

2500

5000

3 hours

6 hours


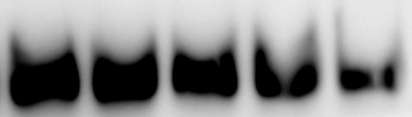

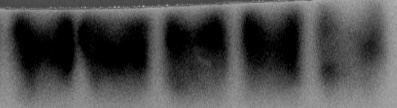


500

1000

2500

5000

10000

24 hours

TIMP1

ACTB

**E**

**D**

**A**

**C**


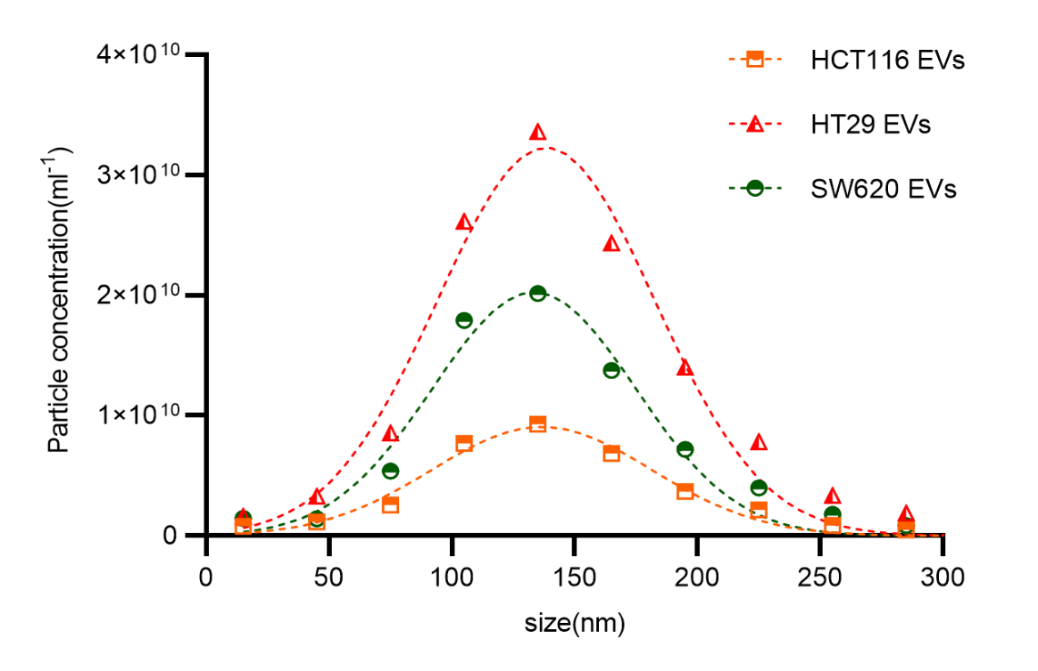

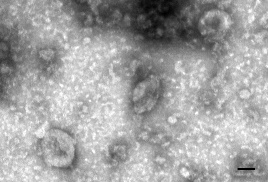


**B**

**Supplementary Fig.S3.** **A** Particle size distribution of the isolated EVs (mean of n=3) of 3 CRC cell lines and representative transmission electron microscopy (TEM) image. **B, C** Densitometry quantification of TIMP1 protein expression normalised to ACTB shown in (Fig 2E, BJ) and (Fig 2E, pF) using imageJ. Recombinant TIMP-1 induces TIMP1 levels in recipient pFs in a time- and concentration-dependent manner. **D** TIMP1 mRNA expression levels in recombinant TIMP1 treated pFs (n=3). **E** Immunoblot of TIMP1 protein levels in the recombinant TIMP1 treated pFs (n=1). **F** Densitometry quantification of TIMP1 protein expression normalised to ACTB shown in (D) using imageJ. Scale bars=100 nm. Error bars depict mean ± SEM. P values were calculated by unpaired t-test: *= p<0.05, ***= p<0.001.

**F**

**A**

**B**

**C**


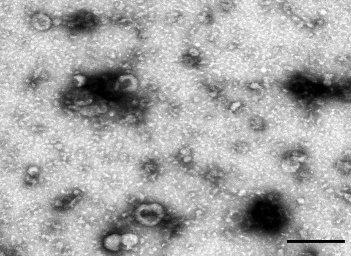

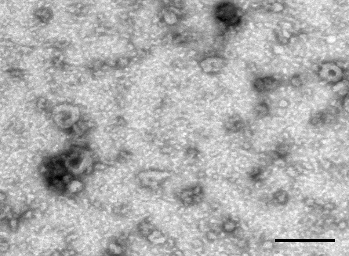

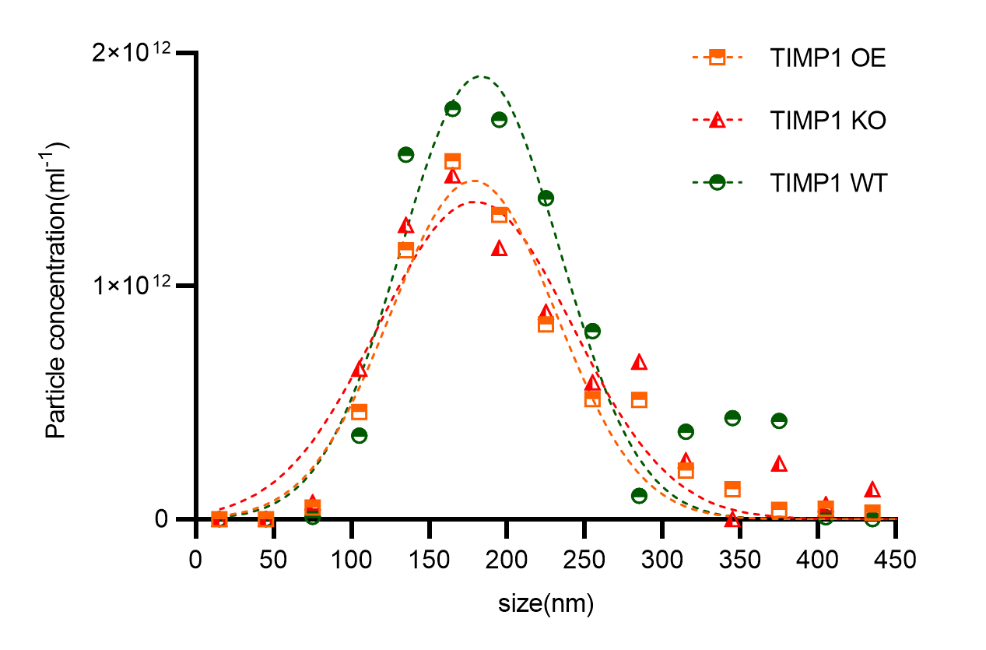


WCL

CD63

ALIX

CALR

CD9

WT

OE1

OE2

WT

KO1

KO2

WT


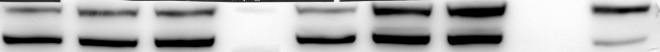

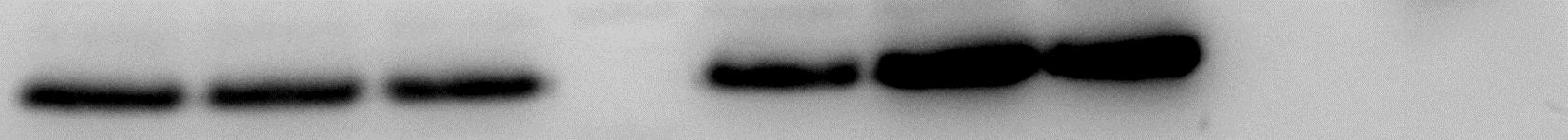

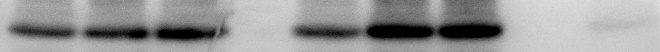


CD81


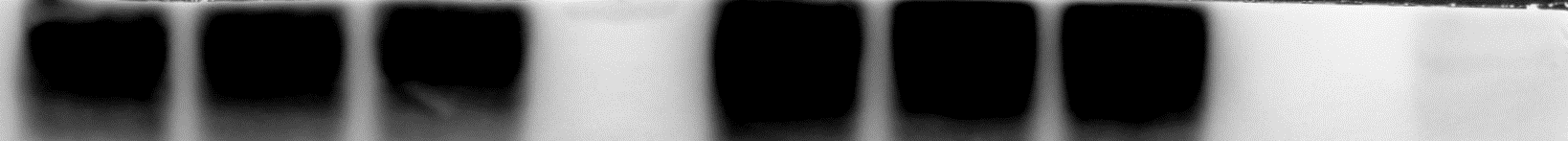

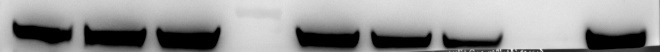

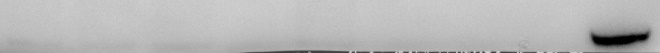


HSP90AA


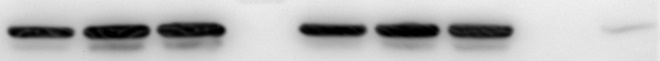


ITGB1


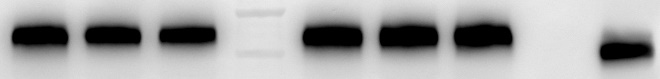


Syntenin-1

TIMP1 OE1

TIMP1 KO1

**Supplementary Fig.S4. Alteration of cellular TIMP1 levels doesn’t affect the EV packaging.**

**A** Nanoparticle tracking analysis (NTA) showing particle size distribution of the isolated EVs (mean of n =3) of TIMP1WT, TIMP1OE and TIMP1KO HCT116 cell lines. **B** Representative image depicts transmission electron microscopy (TEM) of isolated EVs of TIMP1OE and TIMP1KO. **C** Immunoblot analysis of EV proteins ALIX, CD9, CD81, CD63, HSP90AA, Syntenin1, ITGB1, and calreticulin as negative control in EV fraction of modified cell lines. Whole cell lysate (WCL) of HCT 116 TIMP1^WT^ used as internal control. Scale bars=100 nm.


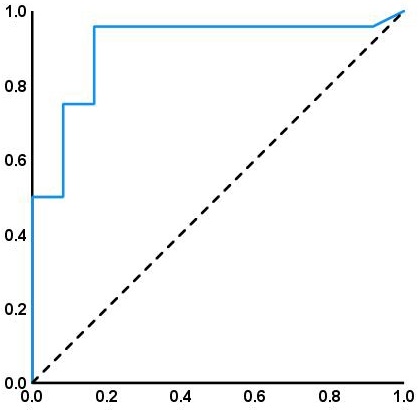


Sensitivity

1-Specificity

**B**

AUC = 0.905

**Supplementary Fig.S5. Serum-derived EVs from CRC patients promotes ECM remodelling. A** Receiver operating characteristics curve (ROC) showing superior stratification power of TIMP1^EV^ (AUC=0.783) in TIMP1 regulation in pFs. **B** ROC curve depicting superior stratification power of TIMP1^EV^ (AUC=0.905) in ECM remodelling. **C** Representative bright field images of collagen matrigel lattices with embedded pFs treated with serum EVs pre-treated with anti-TIMP1 antibody (1 µg) and corresponding untreated controls (n=4 per group), white dashed line highlighting the matrix margins. **D** Percentage of contraction shown in (C) exerted by EV treated pFs in the presence and absence of anti-TIMP1 antibody as calculated using ImageJ. Error bars depict mean ± SEM. P values were calculated by unpaired t test. *=p <0.05, ns= not significant.


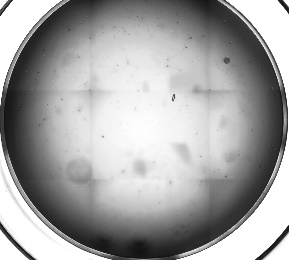

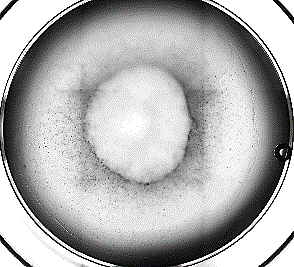

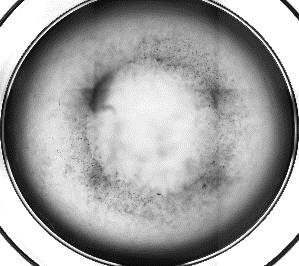

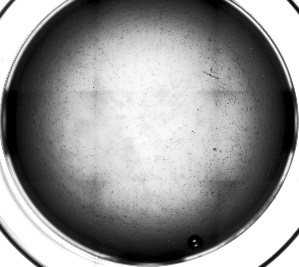

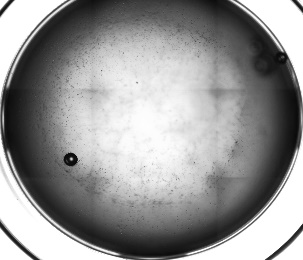

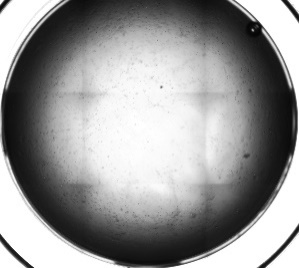


HD

CRC

CRC liver MET

EV

EV+TIMP1 AB


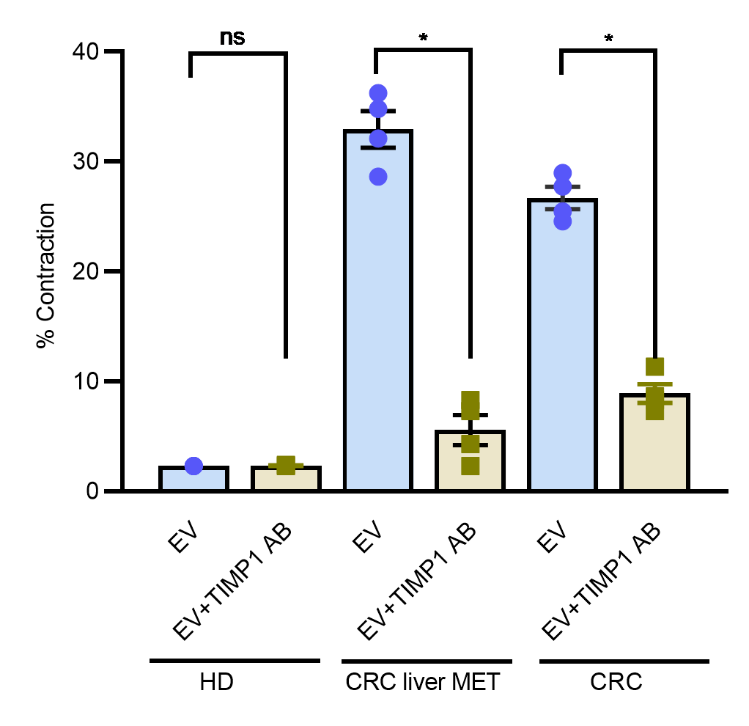


**C**

**D**

**A**


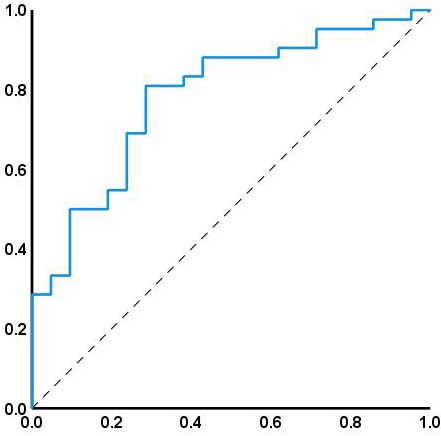


AUC = 0.783

Sensitivity

1-Specificity


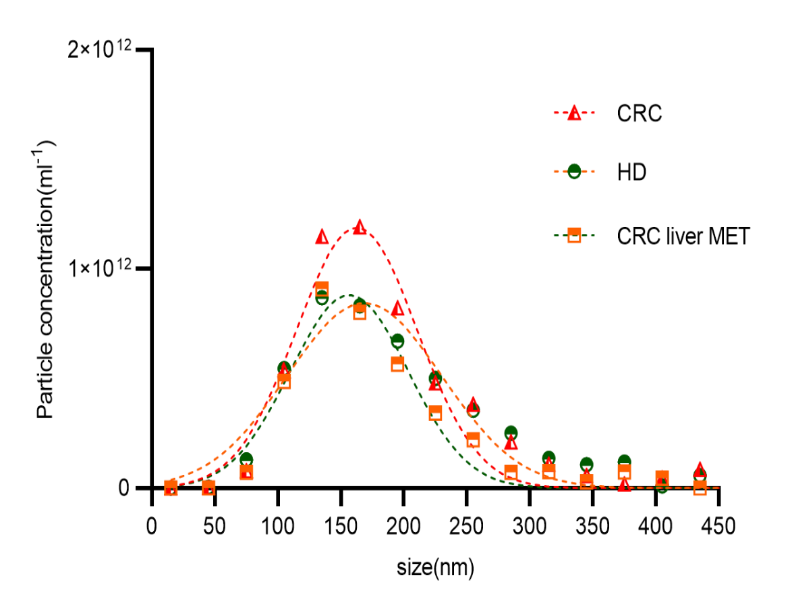


**A**

**C**


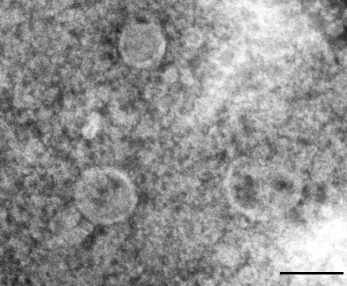


HD


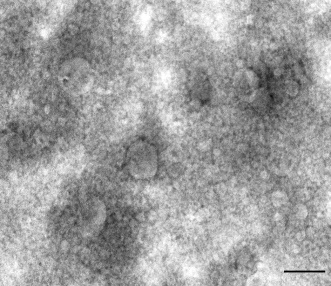


CRC


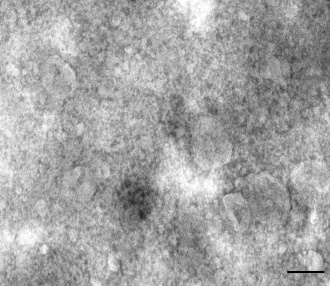


CRC liver MET

**B**


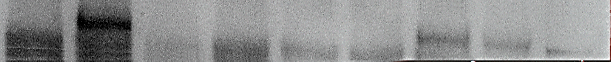


HSP90 AA


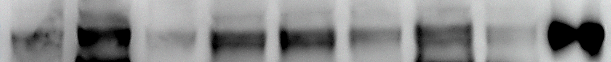


ITGB1


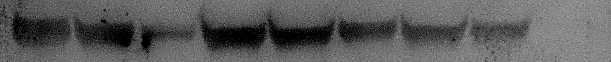


TSG101


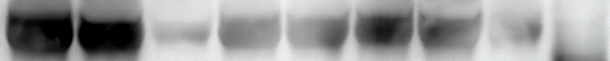


CD63


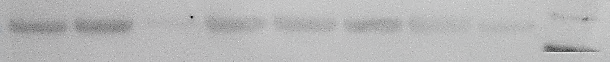


CD81


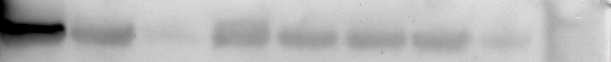


CD9

HD

CRC

CRC liver MET


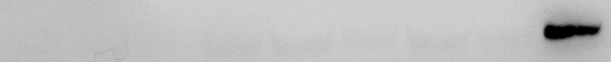


CALR

HCT 116

**Supplementary Fig.S6. Characterisation of serum-derived EVs. A** Nanoparticle tracking analysis (NTA) showing particle size distribution of the isolated EVs (mean of n =3) of Healthy donors (HD), CRC and CRC liver MET. **B** Representative image depicts transmission electron microscopy (TEM) of isolated EVs. **C** Immunoblot analysis of EV proteins CD63, CD9, CD81, HSP90AA, ITGB1, TSG101, and calreticulin as negative control in serum EV fractions. Whole cell lysate (WCL) of HCT 116 cell line used as internal control. Scale bars=100 nm.


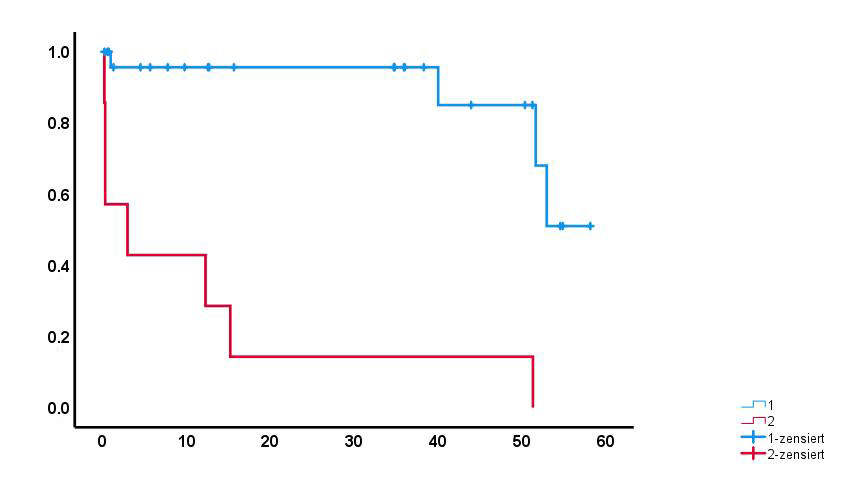


Overall survival rate (%)

p=0.001


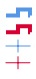


TIMP1^EV^ low

TIMP1^EV^ high

censored

censored

Time(months)

N=26

N=07

Validation cohort II (male)


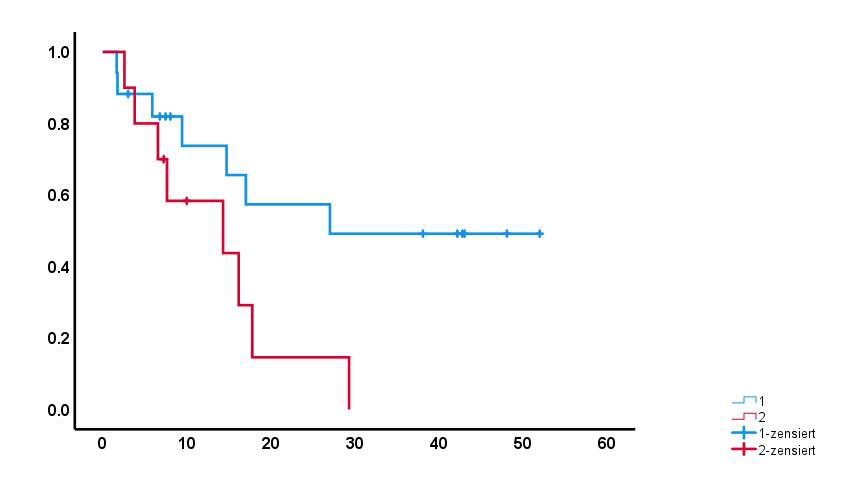

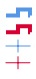


TIMP1^EV^ low

TIMP1^EV^ high

censored

censored

p=0.044

Time(months)

Overall survival rate (%)

N=17

N=10

Validation cohort I (male)

**Supplementary Fig.S7. Gender-based clinical outcome of TIMP1^EV^ in CRC patients. A-C** Kaplan-Meier curve of overall survival of female patients with high or low TIMP1^EV^ in the analysed cohorts. **D-F** Kaplan-Meier curve of overall survival of male patients with high or low TIMP1^EV^ in the analysed cohorts.

**F**

**E**

N=31

N=16


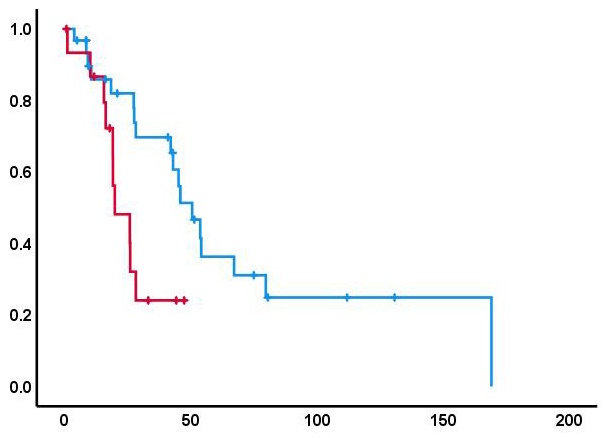

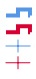


TIMP1^EV^ low

TIMP1^EV^ high

censored

censored

p=0.018

Time (months)

Overall survival rate (%)

Discovery cohort (female)

**A**

Validation cohort I (female)


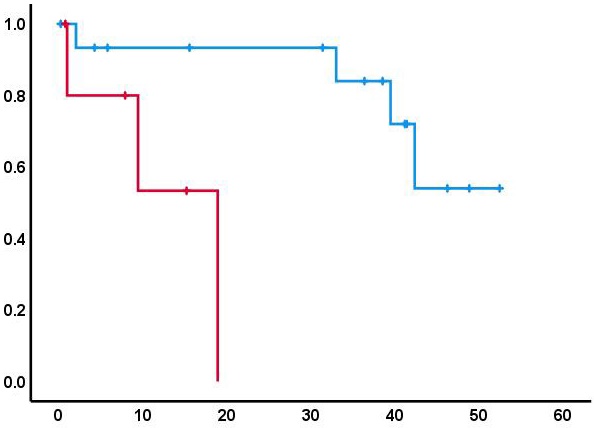


p=0.003

Overall survival rate (%)

Time (months)

N=16

N=06


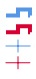


TIMP1^EV^ low

TIMP1^EV^ high

censored

censored

**B**


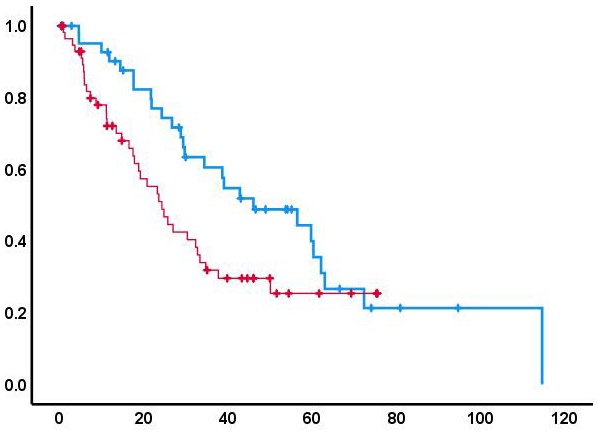


Time (months)

Overall survival rate (%)


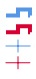


TIMP1^EV^ low

TIMP1^EV^ high

censored

censored

p=0.040

N=45

N=59

Discovery cohort (male)

**D**


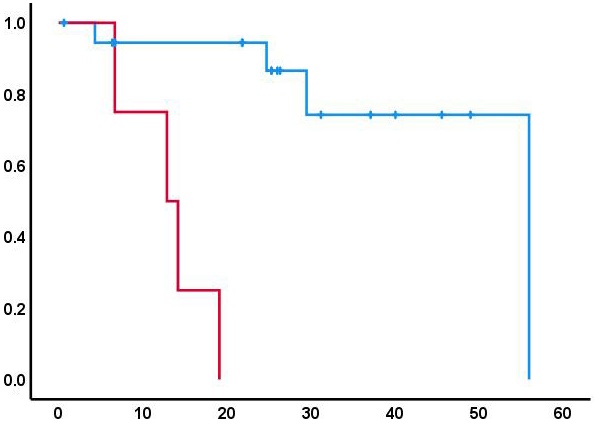

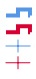


TIMP1^EV^ low

TIMP1^EV^ high

censored

censored

p=0.001

Time (months)

Overall survival rate (%)

N=19

N=04

Validation cohort II (female)

**C**


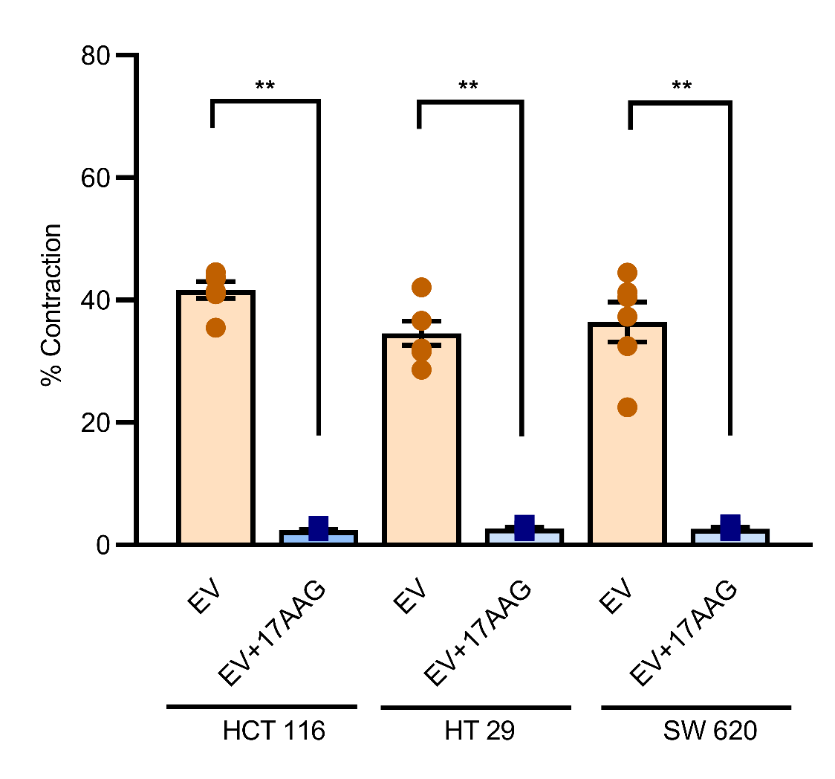


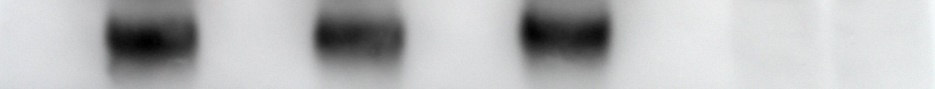

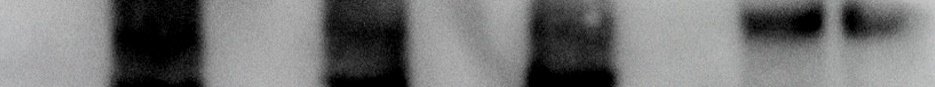

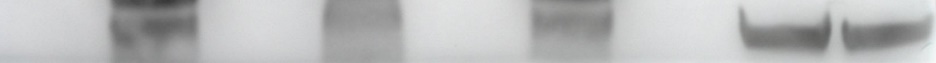


HT29

Input

IP:TIMP1

IgG

HCT 116

HCT116

HT29

SW620

CM

EV

CM

CM

EV

EV


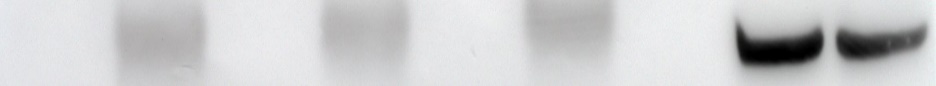


HSP90AA

CD63

TIMP1

ACTB

**A**

**B**


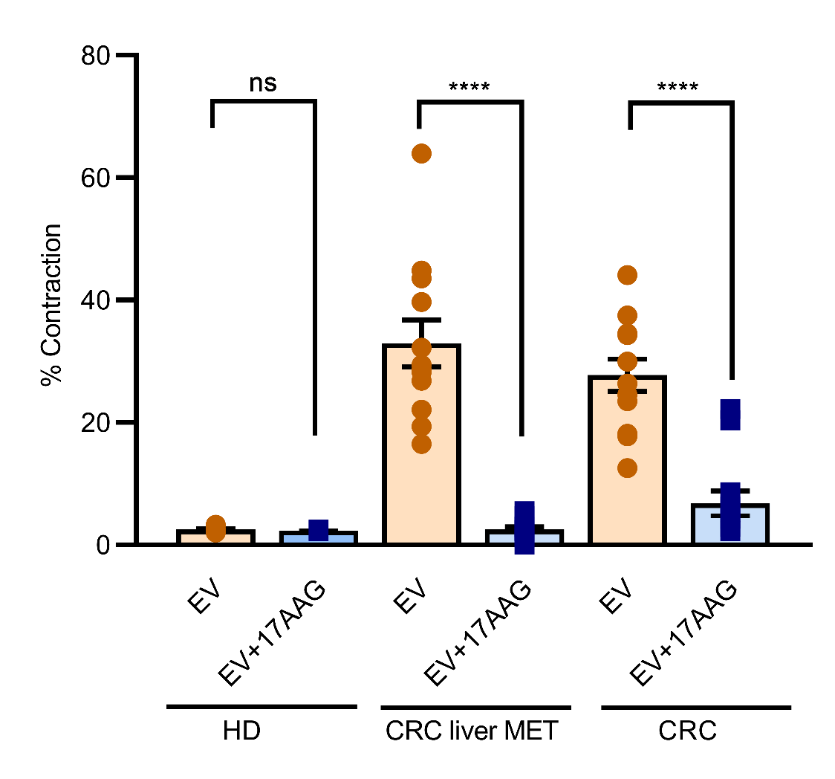


**C**

**D**

**E**


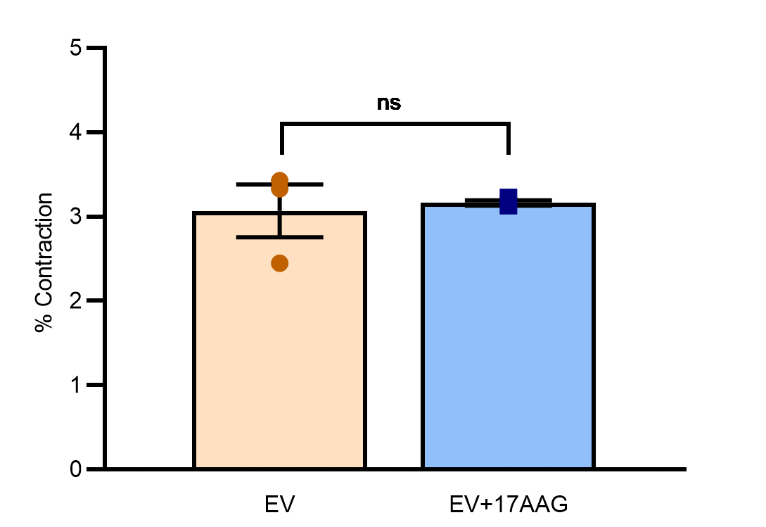


TIMP1^KO^

TIMP1^KO^

+17AAG


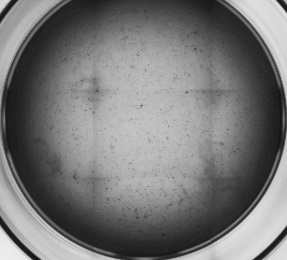

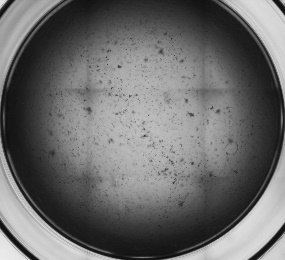


**Supplementary Fig.S8. HSP90 as a target to inhibit TIMP1^EV^ mediated ECM remodelling. A** Immunoblots of CD63, HSP90AA, TIMP1 and ACTB following immunoprecipitation of TIMP1 in pFs treated with EVs and CM from HCT116, HT29 and SW620. WCL input used as internal control. **B** Percentage of contraction shown in (Fig 5F) exerted by EV treated pFs in the presence and absence of 17AAG as calculated using imageJ. **C** Percentage of contraction shown in (Fig 5G) exerted by Serum-EV treated pFs in the presence and absence of 17AAG as calculated using imageJ. **D** Representative bright field images of collagen matrigel lattices with embedded pFs treated with TIMP1^KO^ EVs in the presence and absence of 17AAG (0.3µM), white dashed line highlighting the matrix margins. **E** Percentage of contraction shown in (D) exerted by EV treated pFs in the presence and absence of 17AAG as calculated using ImageJ. All experiments were performed at least 3 times. Error bars depict mean ± SEM. P values were calculated by unpaired t test. **= p<0.01, ****= p<0.0001, ns= not significant.

**Original Data**


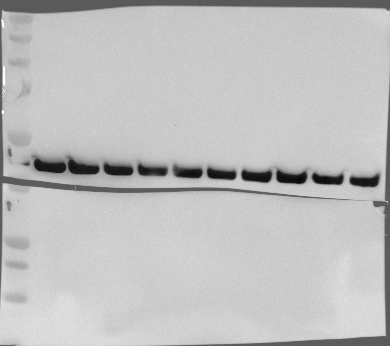

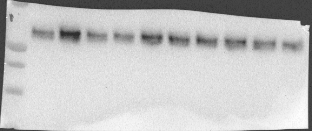

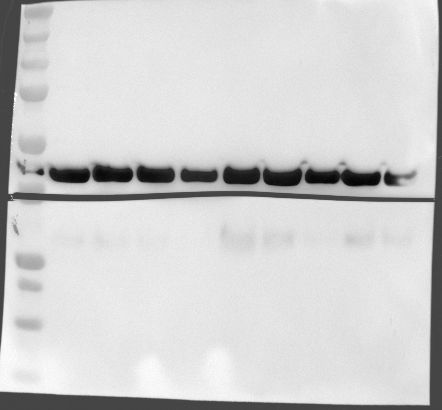

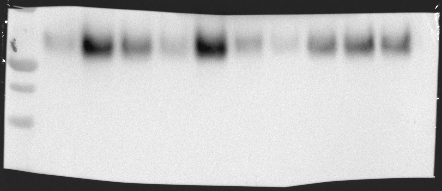

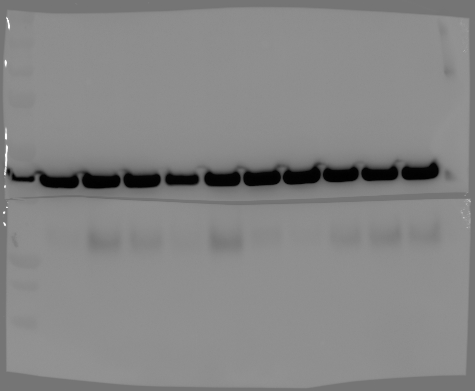

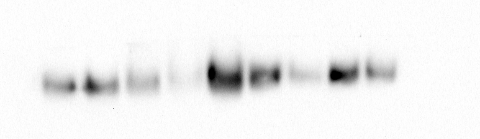


37

50

25

37

50

25

37

50

25

37

50

25

KD

KD

KD

Represented in Figure 2D

Represented in Figure 2D


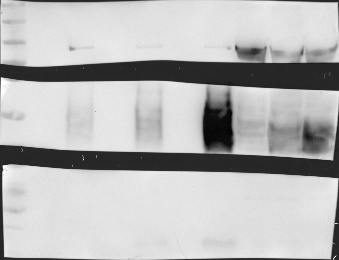

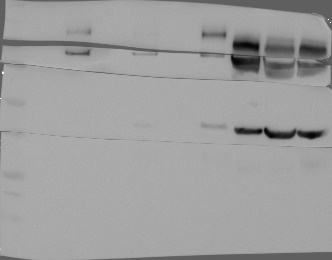


CD63

CD81

ITGB1

HSP90AA

CALR


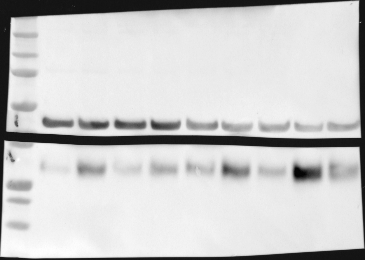

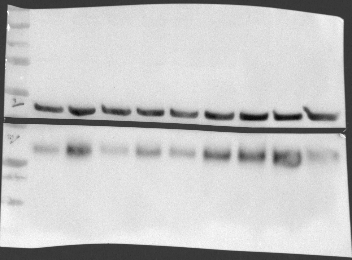


BJ

pF 1

TIMP1

ACTB

Figure 2A


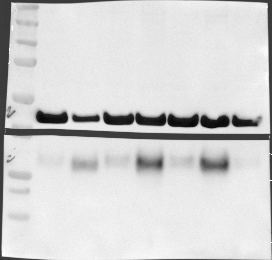


pF 2


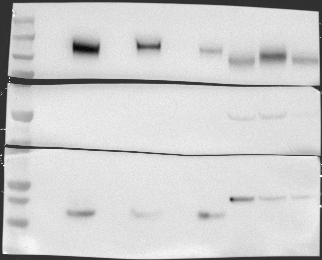


CD 9

CALR

ITGB1


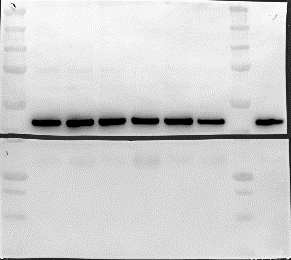

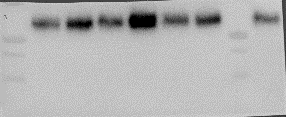


pF 3

Higher exposure

37

50

25


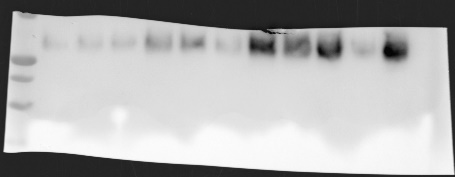

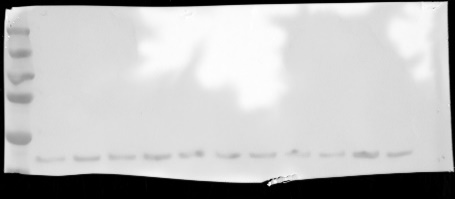

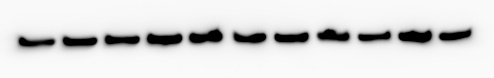

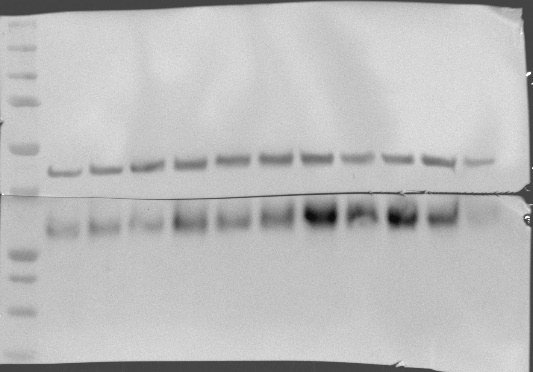

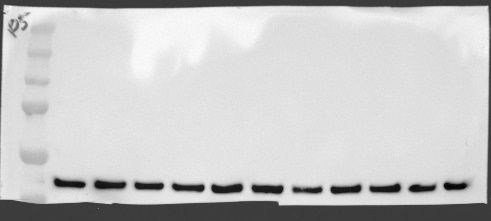

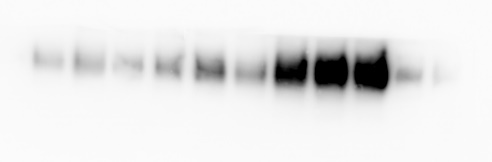

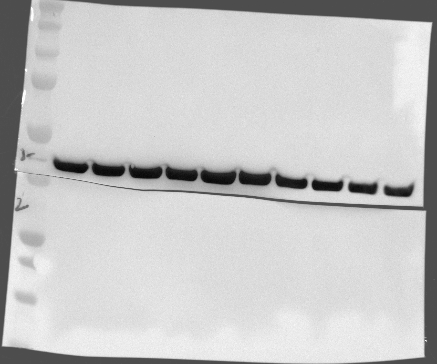

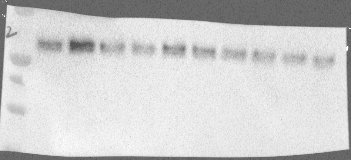


37

50

25

37

50

25

37

50

25

37

50

25

Figure 2B

Figure 2D, Supplemetary 2B-C

BJ

pF

75

100

50

37

100

50

37

75

150

KD

KD

100

50

37

75

150

KD

KD

KD

KD

KD

KD

KD

TIMP1

37

25

Replicate 1

Replicate 2

Replicate 3

Replicate 4

HSP90AA

ACTB

TIMP1

TIMP1^KO^ clones

TIMP1^OE^ clones

37

50

25

75

100

Figure 2E

ITGB1

37

50

75

100

CD63

Syntenin1

25

ALIX

37

50

75

100

TIMP1

CD9

25

20

37

50

75

100

ACTB

25

20

HSP90AA

ACTB

TIMP1

37

50

25

75

100

ALIX

TIMP1

CD9

37

50

25

75

100

20

37

50

75

100

ACTB

25

20

ITGB1

37

50

75

100

CD63

Syntenin1

25

TIMP1^KO^ treated pFs

TIMP1^OE^ treated pFs

Figure 2F-G

HSP90AA

ACTB

TIMP1

37

50

25

75

100

HSP90AA

ACTB

TIMP1

37

50

25

75

100

HSP90AA

ACTB

TIMP1

37

50

25

75

100

Represented in 2G

Represented in Figure 2G

Replicate 1

Replicate 2

Replicate 3

Figure 3F-G

HD

CRC liver MET

CRC

HD

CRC liver MET

CRC

C

HD

CRC liver MET

CRC

C

ACTB

TIMP1

SET 1

SET 2

SET 3

Higher exposure

HD

CRC liver MET

CRC

C

SET 4

HD

CRC liver MET

CRC

C

SET 5

ACTB

TIMP1

Higher exposure

HD

CRC

Higher exposure

CRC liver MET

SET 6

37

50

25

37

50

25

KD

KD

Represented in Figure 3F

Higher exposure

HD

CRC liver MET

CRC

C

SET 10

ACTB

TIMP1

HD

CRC liver MET

CRC

C

SET 7

Higher exposure

HD

CRC liver MET

CRC

Higher exposure

Higher exposure

HD

CRC liver MET

CRC

ACTB

TIMP1

SET 8

SET 9

37

50

25

37

50

25

KD

KD

Figure 5A

TIMP1 IP CRC EVs

CD 63

HSP90AA

37

50

75

100

TIMP1

25

Higher exposure

ACTB

ITGB1

CD9

ACTB

TIMP1

Figure 5B-E

ACTB

TIMP1

HSP90AA AB treated

17AAG treated

37

50

75

37

50

25

37

50

25

Higher exposure

Higher exposure

Higher exposure

25

37

50

25

KD

KD

KD

KD

Represented in Figure 5B

Replicate 1

Replicate 2

ACTB

TIMP1

37

50

25

Higher exposure

Higher exposure

37

50

25

KD

KD

Represented in Figure 5D

Replicate 3

Supplementary Figure 3C

CALR

HSP90AA

CD63

CD9

ITGB1

TIMP1

CD81

150

100

75

50

37

25

15

20

150

100

75

50

37

25

15

20

KD

KD

Higher exposure

150

100

75

50

37

25

15

20

KD

250

Syntenin1

ITGB1

250

Higher exposure

ALIX

HSP90AA

CD63

CD81

ITGB1

TSG101

CD 9

Supplementary Figure 4E

150

100

75

50

37

25

15

20

150

100

75

50

37

25

20

KD

KD

Higher exposure

Higher exposure

CALR

DAY 2

50

37

KD

Higher exposure

Supplementary Figure 5

TIMP1 IP (pFs)

HSP90AA

CD63

37

50

25

75

100

KD

TIMP1

Higher exposure

ACTB
