# Supplementary material for: Extravesicular TIMP-1 is a non-invasive independent prognostic marker and potential therapeutic target in colorectal liver metastases
Source: Oncogene. 2022 Feb 9;41(12):1809–20. doi: 10.1038/s41388-022-02218-9 (PMC8933275; doi:10.1038/s41388-022-02218-9)
Supplement: Supplementary file 2 — Supplementary Table 1-4 [file 41388_2022_2218_MOESM2_ESM.pdf]

Supplementary Table S1

Table S1. Gender-based characteristics of the fibroblast cell lines and EVs used for the study

Characteristics		Fibroblasts			EVs				
					Treatment assays				Biomarker studies
		Overall	BJ	PF	Overall	CRC	CRC liver MET	Cell lines	CRC liver MET
Gender (%)	Female	2 (50)		2 (66.7)	134 (34)	15 (11.2)	26 (19.4)	1 (0.7)	92 (68.7)
	Male	2 (50)	1	1 (33.3)	261 (66)	29 (11.1)	66 (25.3)	2 (0.8)	164 (62.8)

Supplementary Table S2

Table S2. Clinical characteristics of the IHC study cohort

		Overall	CRC liver MET	CRC
n		161	80	81
Median follow-up time (days)		2945	1581	1364
Age (median)		141	68.5	72.5
Gender (%)	Female	76 (47.2)	52 (65)	24 (29.62)
	Male	85 (52.8)	28 (35)	57 (70.37)
Residual disease (%)	No	152 (94.41)	75 (93.75)	77 (95.1)
	Yes	9 (11.11)	5 (6.25)	4 (4.94)
Neoadjuvant therapy (%)	No	110 (68.32)	50 (62.5)	60 (74.07)
	Yes	51 (31.68)	30 (37.5)	21 (25.92)
Tumor differentiation (%)	G1-G3	101 (62.73)	49 (61.25)	52 (64.2)
	G4	2 (1.24)	1 (1.25)	1 (1.23)
TNM (%)	I-II	48 (29.81)	6 (7.5)	42 (51.9)
	III-IV	104 (64.6)	71 (88.75)	33 (40.74)
KRAS (%)	Mutant	28 (17.39)	18 (22.5)	10 (12.35)
	Wild type	9 (5.59)	8 (10)	1 (1.23)

Supplementary Table S3

Table S3. Clinical characteristics of the ELISA study cohort

		Overall	Discovery Cohort	Validation Cohort I	Validation cohort II (external)
n		256	151	49	56
Median follow-up time (days)		1728	753	310	665
Age (median)		66	65	69	68
Gender (%)	Female	92 (35.9)	47 (51.1)	22 (23.9)	23 (25.0)
	Male	164 (64.1)	104 (63.4)	27 (16.5)	33 (20.1)
Neoadjuvant therapy (%)	No	68 (30)	46 (67.6)	10 (14.7)	12 (17.6)
	Yes	159 (70)	105(66)	39 (24.5)	15 (9.4)
TNM (%)	IVA	182 (78.8)	100 (54.9)	32 (17.6)	50 (24.5)
	IVB	49 (21.2)	39 (79.6)	4 (8.2)	6 (12.2)
KRAS (%)	Mutant	66 (46.5)	51 (77.3)	15 (22.7)	0 (0.0)
	Wildtype	76 (53.5)	63 (82.9)	13 (17.1)	0 (0.0)

Supplementary Table S4

Primer list

Gene	Primer sequence	Final concentration (pmol/ul)	Application
ACTA2	CATCGGAAATGAACGTTTCC	1	RT-qPCR
	CCCCTGATAGGACATTGTTAGC		
COL1A1	GTGCTAAAGGTGCCAATGGT	1	RT-qPCR
	ACCAGGTTCACCGCTGTTAC		
FN1	CAAGCATGTCTCTCTGCCAAG	1	RT-qPCR
	CAGAACAGGCAATGTGCAGC		
TIMP1	TGTTGCTGTGGCTGATAG	1	RT-qPCR
	CTGGTATAAGGTGGTCTGG		
TIMP2	ACGATATACAGGCACATTATG	3	RT-qPCR
	GGTCAGGAGTCTTAACAGG		
TIMP3	GGTGAAGCCTCGGTACATCT	1	RT-qPCR
	AGGACGCCTTCTGCAACTC		
TIMP4	TTTCTTCTGGCTTAGTCTGTTTTCT	1	RT-qPCR
	ATTCGCCATTTCTCCCCTACCA		
TAGLN	AAGAATGATGGGCACTACCG	1	RT-qPCR
	ATGACATGCTTTCCCTCCTG		
VTN	GAGTGCAAGCCCCAAGTGAC	1	RT-qPCR
	GCCATCGTCATAGACCGTGT		
TNC	TAACGGTGGTGGATTCTGGG	1	RT-qPCR
	CTTCCGGTTCGGCTTCTGTA		
ACTB	CATGTACGTTGCTATCCAGGC	1	RT-qPCR
	CTCCTTAATGTCACGCACGAT		
NRP1	CCCAACAGCCTTGAATGCAC	1	RT-qPCR
	ATTTCTAGCCGGTCGTAGCG		
NRP2	CTGTGGGTCATCCGTGAGGAC	1	RT-qPCR
	ATGGGTTCCATGCAGTTCTCCAG		

Application	Primer name	Primer sequence
TIMP1 knockout	TIMP1 gRNA KO	CAGGTCCCACAACCGCAGCG

Antibody List

Application	Antibody/ Protein recognized	Vendor	Product reference	Antibody dilution
Immunoblot	TIMP-1 (D10E6)	Cell Signaling	D10E6	1:1000
	TSG101 (4A10)	abcam	ab83	1:500
	CD81	Santa Cruz biotechnology	sc-166029	1:1000
	Integrin-ß1	Cell Signaling	9699	1:1000
	anti-TSG101	abcam	ab83	1:500
	HSP90AA	Enzo Life Tech.	ADI-SPS-771-D	1:1000
	Anti-CD63 antibody	abcam	68418	1:500
	Syntenin1(EPR8102)	abcam	ab133267	1:1000
	Anti-ALIX antibody [3A9]	abcam	ab117600	1:1000
	Calreticulin	Cell Signaling	2891S	1:1000
	anti-mouse IgG HRP-linked	Cell Signaling	7076	1:5000
	anti-rabbit IgG HRP linked	Cell Signaling	7074	1:3000
Immunoprecipitation				
	HSP90AA	LS Bio	LS-B7446-50	1:1000
	Anti-CD63 antibody	Novus biologicals	LAMP3/2788	1:1000
Immunohistochemistry	TIMP-1 (D10E6)	Cell Signaling	D10E6	1:200

Inhibitors

Product name	Vendor	Product reference	Dilution
Heparin ammonium salt from porcine intestinal mucosa ≥180 USP units/mg	Merck-Sigma Aldrich	H7484	100µg/ml
17AAG-CAS 75747-14-7 - Calbiochem	Sigma- Aldrich	75747-14-7	0.5µM
